# Multi-omics analysis dissects the genetic architecture of seed coat content in *Brassica napus*

**DOI:** 10.1186/s13059-022-02647-5

**Published:** 2022-03-28

**Authors:** Yuting Zhang, Hui Zhang, Hu Zhao, Yefan Xia, Xiangbo Zheng, Ruyi Fan, Zengdong Tan, Chenhua Duan, Yansong Fu, Long Li, Jiang Ye, Shan Tang, Honghong Hu, Weibo Xie, Xuan Yao, Liang Guo

**Affiliations:** 1 National Key Laboratory of Crop Genetic Improvement, Huazhong Agricultural University, Wuhan, China; 2 Hubei Hongshan Laboratory, Wuhan, China; 3 Hubei Key Laboratory of Agricultural Bioinformatics, College of Informatics, Huazhong Agricultural University, Wuhan, China

**Keywords:** *Brassica napus*, Seed coat content, Seed oil content, Co-expression network, eQTL, TWAS, Phenylpropane pathway

## Abstract

**Background:**

*Brassica napus* is an important vegetable oil source worldwide. Seed coat content is a complex quantitative trait that negatively correlates with the seed oil content in *B. napus*.

**Results:**

Here we provide insights into the genetic basis of natural variation of seed coat content by transcriptome-wide association studies (TWAS) and genome-wide association studies (GWAS) using 382 *B. napus* accessions. By population transcriptomic analysis, we identify more than 700 genes and four gene modules that are significantly associated with seed coat content. We also characterize three reliable quantitative trait loci (QTLs) controlling seed coat content by GWAS. Combining TWAS and correlation networks of seed coat content-related gene modules, we find that *BnaC07.CCR-LIKE* (*CCRL*) and *BnaTT8s* play key roles in the determination of the trait by modulating lignin biosynthesis. By expression GWAS analysis, we identify a regulatory hotspot on chromosome A09, which is involved in controlling seed coat content through *BnaC07.CCRL* and *BnaTT8s*. We then predict the downstream genes regulated by *BnaTT8s* using multi-omics datasets. We further experimentally validate that *BnaCCRL* and *BnaTT8* positively regulate seed coat content and lignin content. *BnaCCRL* represents a novel identified gene involved in seed coat development. Furthermore, we also predict the key genes regulating carbon allocation between phenylpropane compounds and oil during seed development in *B. napus*.

**Conclusions:**

This study helps us to better understand the complex machinery of seed coat development and provides a genetic resource for genetic improvement of seed coat content in *B. napus* breeding.

**Supplementary Information:**

The online version contains supplementary material available at 10.1186/s13059-022-02647-5.

## Background

*Brassica napus* is one of the most important oil crops which produces approximately 13% of the edible oil worldwide (USDA ERS, 2020). The seeds of *B. napus* are mainly composed of two parts, embryo and seed coat [1]. Though oil and protein are concentrated in the embryo, seed coat plays crucial roles in many biological processes such as signal transmission, nutrition transportation, protection against biotic and abiotic stresses, and control of seed size [2, 3]. The seed coat is derived from the outer and inner integuments of the ovule [4]. Proanthocyanidins (PAs), an end products of flavonoid biosynthesis, are synthesized and accumulated in inner integument during the seed development [5]. The innermost cells of the outer integument form a palisade layer, which shows cell wall thickenings in the seed coats of *B. napus* [6, 7]. Seed coat content (SCC) refers to the ratio of seed coat mass to total seed weight and it is directly affected by the thickness of the seed coat, which is controlled by secondary cell wall formation, including the biosynthesis of cellulose, hemicellulose, and lignin [8, 9].

Previous studies have shown that seed oil content (SOC) is significantly negatively correlated with the SCC in *B. napus* [10, 11]. Several co-located QTLs were found to control SCC, SOC, and seed coat color [12]. More interestingly, malonyl-CoA is not only the substrate for fatty acid synthesis, but also a substrate for flavonoid synthesis [13]. In addition, malonyl-CoA is produced from acetyl-CoA, which is also involved in the synthesis of cell wall xyloglucan [14]. The seed coat color of *B. napus* is determined by the content of PAs (epicatechin and its derivatives) and insoluble PAs (In-PAs) [15–17]. It has been revealed that the of content PAs and polyphenol was higher in the seed coat of the black-seeded variety compared with the yellow-seeded variety [17]. Yellow-seeded rape has the advantages of thin seed coat, low seed coat and lignin content, high oil and protein content, and transparent oil [18–20]. Therefore, the development of yellow-seeded cultivars has been an important breeding goal for *B. napus* [21–23]. Nevertheless, the reason for the correlation between seed coat color and some traits of *B. napus* with yellow seeds such as lower SCC and higher SOC has not yet been fully elucidated. A full understanding of the metabolite synthesis in the seed coat which affects SCC would be beneficial for further improvement of SOC of *B. napus* [24–26].

Lignin, a complex natural polymer of monolignols, is one of the main components of secondary cell walls. Low lignin (acid detergent lignin) content is related to reduce of seed coat thickness [27, 28]. Both lignin and flavonoids are synthesized by the phenylpropane pathway, in which phenylalanine ammonia-lyase (PAL), trans-cinnamate 4-monooxygenase (C4H), and 4-coumarate-CoA ligase (4CL) catalyze phenylalanine to coumaroyl-CoA step by step and the lignin and flavonoids are finally produced in different branch pathways, respectively [29]. A close correlation between the lignin mainly located in seed coat and seed color was found in *B. napus* [30]. Some QTLs for lignin content and seed coat color were also found to co-locate in *B. napus* [31, 32]. Lignin and pigment synthesis share common precursors such as coumaroyl-CoA in the phenylpropane biosynthesis pathway may explain the reason for the coincidence of light seed color and low lignin content [33]. Therefore, lignin biosynthesis and flavonoid accumulation in the seed coat are likely to be regulated by the same key genes. More recent studies have reported that seed lignin content significantly positively correlates with SCC and negatively correlates with SOC and reduction of lignin content results in an increase of SOC [27, 34–36]. However, the genetic basis of SCC during the seed coat formation in *B. napus* remains unclear.

With the development of omics technologies, the use of omics data in resolving regulatory mechanisms of complex traits is becoming an important tool [37–40]. GWAS has been widely used in various crops such as rice, maize, cotton, and rapeseed, as it can locate the loci through the association of markers with traits [41–52]. Recently, expression genome-wide association analysis (eGWAS), co-expression module analysis and transcriptome-wide association studies (TWAS) based on population transcriptome data have been used to further reveal the regulatory relationship between gene and gene or phenotype [53–61]. In crops, several studies have been conducted to explore the trait-related regulatory genes and modules by the population transcriptome data with proven success [62, 63].

To dissect the genetic basis of SCC in *B. napus*, we attempted to identify genes, QTLs, and regulatory networks associated with SCC of *B. napus* using TWAS, GWAS, and gene module analysis in this study. We identified a great number of significant genes, loci, and four gene modules which are potentially involved in the regulation of SCC during seed coat formation in *B. napus.* The identification of SCC-related genes, loci, and regulatory networks would enrich the knowledge of genetic basis of SCC trait and accelerate the breeding *B. napus* varieties with low SCC and high SOC.

## Results

### Transcriptome-wide association studies illuminate the molecular basis of natural variation of SCC

**Fig. 1:**
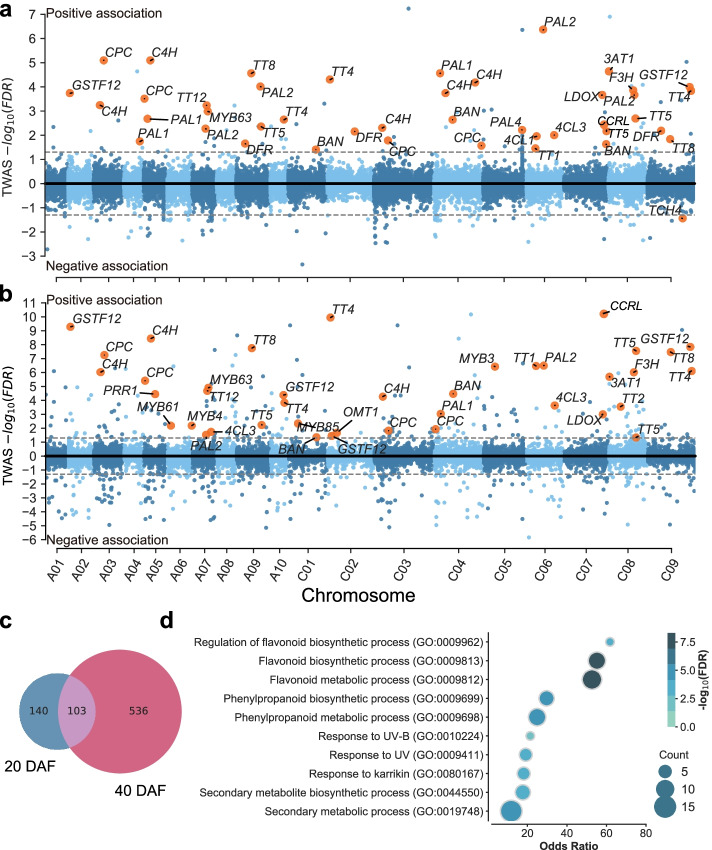
Transcriptome-wide association studies of SCC. **a**, **b** Manhattan plot of TWAS results (FDR < 0.05) for SCC at 20 DAF (**a**) and 40 DAF (**b**). Each point represents a single gene tested. Genomic positions of genes are plotted on the *X*-axis and the log-transformed FDR values of association between gene expression and SCC are plotted on the *Y*-axis. The genes positively or negatively associated with the SCC are plotted above or under the black bold line, respectively. The golden dots indicate genes involved in the phenylpropanoid biosynthesis pathway and secondary cell wall development identified in Arabidopsis. The dashed gray horizontal lines represent the significance level. **c** Venn diagram showing shared genes between significant genes identified by TWAS at 20 DAF and 40 DAF. **d** GO enrichment analysis of overlapped significant genes identified by TWAS at 20 DAF and 40 DAF. The dot size and color indicate the gene number and the range of FDR values, respectively

We obtained SCC phenotypic data of 382 *B. napus* accessions and the results revealed a large variation in SCC ranging from 11.83 to 21.87% (Additional file 2: Table S1; Additional file 1: Fig. S1a). We found a significant negative correlation between SCC and SOC (square of Pearson coefficient = 0.18, *P* = 3.33×10^−19^. Additional file 1: Fig. S1b). To further determine the genes involved in the determination of SCC, TWAS were performed using transcriptomic data from the developing seeds of 257 accessions at 20 days after flowering (DAF) DAF and 253 accessions at 40 DAF collected from data in our previous studies (Additional file 2: Table S2) [63], because seeds oil biosynthesis starts from early developmental stage and seed oil is rapidly accumulated in late developmental stage [64]. There were 243 and 639 genes significantly associated with SCC at 20 DAF and 40 DAF, respectively (Fig. 1a, b; Additional file 2: Table S3, 4). Moreover, 103 significant genes were detected in both stages of seed development (Fig. 1c; Additional file 2: Table S5). A large proportion of TWAS-significant genes were shown to be related to phenylpropane and flavonoid biosynthesis pathways, such as *PAL*, *4CL*, *C4H*, *BANYULS* (*BAN*), *dihydroflavonol-4-reductase* (*DFR*), *flavanone 3-hydroxylase* (*F3H*), and genes involved in *TRANSPARENT TESTA* (*TT*) family and MYB family genes (odds ratio = 724.85, *P* = 2.05×10^−8^) [65, 66]. *TT* family genes have been shown to participate in the PAs synthesis in Arabidopsis, we located multiple homologous genes of *TT2*, *TT4*, *TT5*, *TT8*, and *TT12* in *B. napus*, indicating that the synthesis of PAs shows a close relationship with SCC, consistent with the previous results reported by Yan et al. [12] and Stein et al. [67]. In addition to *PAL*, *C4H*, and *4CL* involved in the general phenylpropanoid pathway, it is worth to note that genes related to lignin biosynthesis, such as MYB family transcription factor genes *MYB85*, *MYB63*, *MYB7* and *MYB4* [68], as well as genes encoding catalytic enzymes such as *pinoresinol reductase 1* (*PRR1*), *caffeic acid O-methyltransferase 1* (*OMT1*), *beta-glucosidase 46* (*BGLU46*) [69–71], and *Cinnamoyl-CoA reductase-like* (*CCRL*) [72–74] were also present in the TWAS-significant genes, indicating that SCC is affected by lignin biosynthesis (Additional file 2: Table S6, 7). The discovery of genes related to the secondary wall, such as *MYB61* and *xyloglucan endotransglucosylase* (*TCH4*) also showed that the effect of secondary wall metabolism on SCC cannot be underestimated [75, 76]. These results indicate that there is a potential correlation between SCC and the phenylpropane biosynthesis and metabolic pathway or SOC, which is consistent with previous studies [4, 12].

We further performed GO enrichment analysis of significant genes (FDR < 0.05) identified by TWAS at 20 DAF, 40 DAF, and the 103 overlapped significant genes in two stages, respectively. The results showed that these genes were mainly enriched in the biosynthetic pathways of secondary metabolites such as flavonoids and lignin. In addition, some genes were involved in stress-related and other processes (Fig. 1d; Additional file 1: Fig. S2; Additional file 2: Table S8-10). Taken together, these results indicate that the variation of SCC may be mainly determined by the secondary metabolites synthesized through the phenylpropane pathway.

### Four gene modules are involved in controlling SCC in *B. napus*

**Fig. 2:**
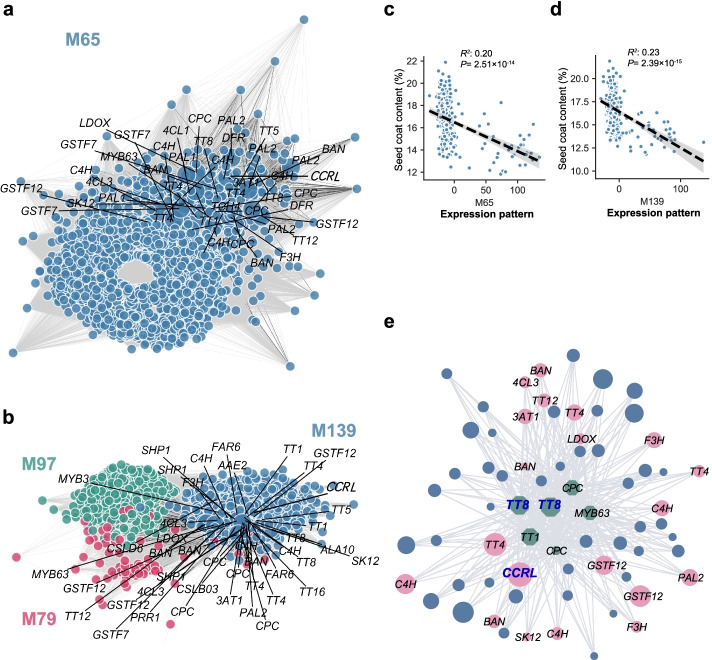
SCC-related gene modules and co-expression networks in seeds. **a** Gene correlation network of SCC-related module (M65) at 20 DAF. Phenylpropanoid biosynthesis-related genes significantly associated to SCC detected by TWAS at 20 DAF are labeled in the module. **b** Gene correlation network of SCC-related modules (M97, M79, M139) at 40 DAF. Phenylpropanoid biosynthesis-related genes significantly associated to SCC detected by TWAS at 40 DAF are labeled in the module. **c** Correlation between the expression pattern (*X*-axis) of M65 and SCC (*Y*-axis). **d** Correlation between the expression pattern (*X*-axis) of M139 and SCC (*Y*-axis). **e** Correlation network built on the overlapped genes of the TWAS-significant genes, M65 and M139. The green dots represent phenylpropanoid biosynthesis-related transcription factors and red dots represent other phenylpropanoid biosynthesis-related genes, the blue dots represent the rest of genes. The size of a node is proportional to its degree (the number of correlated genes). Genes in **a**, **b**, and **e** are shown by dots and gene-gene correlations (FDR < 0.01) are indicated by gray lines

To further reveal how multiple genes coordinate to regulate SCC, we analyzed the gene modules identified in two developmental stages of *B. napus* seeds to elucidate the putative regulatory mechanisms of SCC. At 20 DAF, only one significant component (the corresponding gene module was termed M65) was found to be significantly correlated with SCC (M65: *LMM P* = 1.52×10^−11^) (Fig. 2a). At 40 DAF, three components (the corresponding gene module was termed M139, M97, and M79) were significantly correlated with SCC (M139: *LMM P* = 6.09×10^−13^, M79: *LMM P* = 1.14×10^−7^, M97: *LMM P* = 4.1×10^−4^) (Fig. 2b). Except for M97, the other three modules were found to be significantly correlated with SOC in Tang’s study [63]. The genes in these modules are involved in transcriptional regulation, stress response, signal transduction, transmembrane transport, and photosynthesis. We performed correlation analysis between the modules and SCC and found that only M65 and M139 were significantly negatively correlated with SCC at both seed developmental stages (Fig. 2c, d).

To find the key regulators of seed coat development, the 68 genes overlapped among TWAS-significant genes, M65 at 20 DAF and M139 at 40 DAF were further analyzed. It was found that BnaC06g08390D (*TT1*), BnaA02g31540D (*WRKY50*), BnaA07g20440D (*MYB63*), BnaA05g01400D (*CPC*), DnaC09g24870D (*CPC*), BnaC03g25810D (*CPC*), BnaC09g24870D (*TT8*), and BnaA09g22810D (*TT8*) in the gene set are transcription factors associated with the phenylpropane biosynthesis and expression of the two *TT8* homologous genes among them exhibited the highest association with SCC. In addition, we also identified BnaC07g42660D (*CCRL*), BnaC02g05070D (*TT4*), BnaA03g60670D (*BAN*), BnaC08g22640D (*F3H*), BnaC06g14510D (*PAL2*), BnaA02g03440D (*GSTF12*), and BnaA05g11950D (*C4H*), which are also phenylpropane biosynthesis pathway-related genes, among all genes a *CCRL* gene showed the highest association with SCC (Fig. 2e). TT8 is a well-known bHLH transcription factor regulating flavonoid synthesis [77] and *CCRL* encodes a NAD(P)-binding Rossmann-fold superfamily protein, which was predicted to participate in lignin synthesis as a cinnamoyl-CoA-reductase like protein [78, 79]. We therefore considered *BnaTT8s* and *BnaC07.CCRL* as the candidate key regulators of the SCC trait for further study.

### *qSCC.A09* infers a regulatory hotspot of SCC

**Fig. 3:**
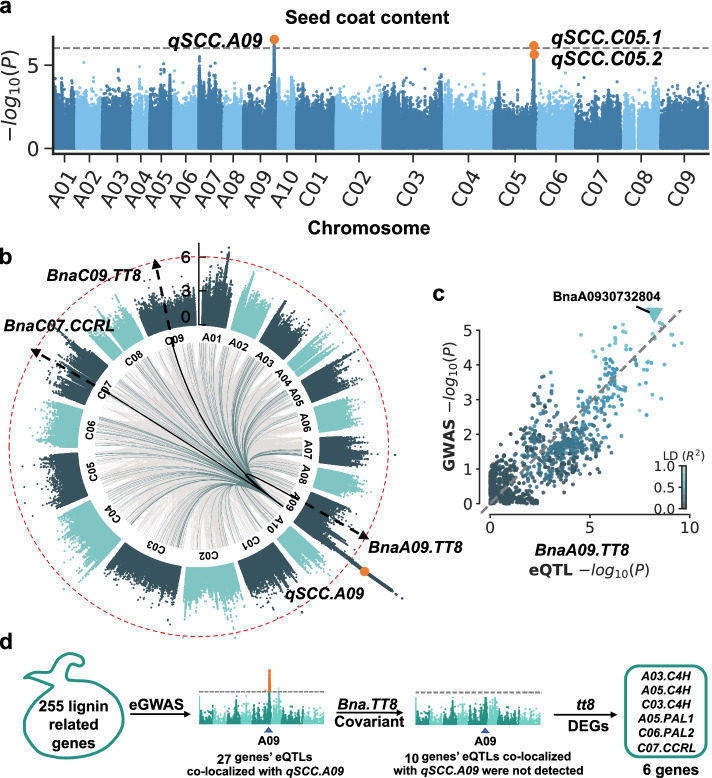
The regulatory hotspot on chromosome A09 affects SCC. **a** Manhattan plot of GWAS results for SCC. Three significant QTLs are denoted. **b** Manhattan plot of GWAS results for expression value of *BnaA09.TT8*, *BnaC09.TT8* and *BnaC07.CCRL*. The SCC QTLs *qSCC.A09* identified by GWAS are denoted. Significant eGWAS associations for TWAS-significant genes at 40 DAF are marked (gray lines); associations co-localized with *qSCC.A09* are highlighted in green. *BnaA09.TT8*, *BnaC09.TT8* and *BnaC07.CCRL* localization results are highlighted in black. **c** The log-transformed *P* values form GWAS plotted against those for variants from eGWAS of *qTT8.A09* (around *qSCC.A09* lead SNP 150 kb). **d** Flowchart of screening of putative phenylpropanoid and lignin synthesis-related genes regulated by *BnaTT8s*. Based on the eGWAS results of phenylpropanoid and lignin synthesis-related genes, whose eQTLs co-localized with *qSCC.A09* were detected. eQTLs of some genes were not detected to be co-localized with *qSCC.A09* using *BnaTT8s* as a covariate. Among these genes, differentially expressed genes when *BnaTT8s* were mutated were identified

GWAS were performed for SCC trait and 3 significant QTLs were identified, two of them were located on chromosome C05 named as *qSCC.C05.1* and *qSCC.C05.2*, and one was on chromosome A09 named as *qSCC.A09* (Fig. 3a). A series of genes related to seed coat development, such as *mucilage-related 21* (*MUCI21*), *PAL4*, *subunit of exocyst complex 8* (*SEC8*), and *MYB52*, were located in three QTL regions (Additional file 2: Table S11) and there were multiple TWAS-significant genes located in the three SCC-related QTL regions (Additional file 2: Table S12).

eGWAS is a powerful method to localize expression quantitative trait loci (eQTLs) that function upstream of target genes [53–55]. We subsequently performed eGWAS on *BnaC07.CCRL* and *BnaTT8s* to identify its eQTLs. Interestingly, we found three eQTLs *qTT8.A09*, *qTT8.C09*, and *qCCRL.C07* identified by eGWAS co-located with *qSCC.A09* (Fig. 3b, c; Additional file 2: Table S13). In addition, we performed eGWAS for 639 TWAS-significant genes at 40 DAF and found that over 500 TWAS significant genes associated with SOC had eQTLs localized in the *qTT8.A09* regulatory hotspot (Additional file 1: Fig. S3; Additional file 2: Table S14). GO enrichment analysis of the 243 genes revealed a significant bias in many categories associated with phenylpropanoid metabolism, abiotic stresses, such as responses to UV, chemical, and wounding (Additional file 2: Table S15). Thus, these results suggest that the hotspot on chromosome A09 can regulate not only *BnaC07.CCRL* and *BnaTT8s* but also a series of TWAS-significant genes.

To further investigate whether the *qSCC.A09* hotspot region could regulate the expression of lignin-related genes through transcription factor *BnaTT8*, a total of 255 *B. napus* homologous genes related to lignin biosynthesis were identified and eGWAS were performed for these genes (Additional file 2: Table S16). The eQTLs for 27 of these genes were co-localized in the *qSCC.A09* hotspot region, indicating that they are potentially regulated by *qSCC.A09*. However, when adding the expression of *BnaTT8* as a covariate to the eGWAS analysis, the eQTLs of 10 phenylpropanoid biosynthesis-related genes could not be detected, including *BnaA03.C4H*, *BnaA05.PAL1*, *BnaA05.C4H*, *BnaA07.MYB63*, *BnaC03.MYB36*, *BnaC03.C4H*, *BnaC03.4CL*, *BnaC03.CCoAOMT*, *BnaC06.PAL2*, and *BnaC07.CCRL* (Additional file 2: Table S17). Among them, the *C4H* and *PAL* homologs and *CCRL*, are differentially expressed in the *BnaTT8* mutant compared with WT [80] (Fig. 3d). Furthermore, the expression levels of these six genes showed significant positive correlation with *BnaTT8* expression (Additional file 1: Fig. S4, 5), indicating that the *qSCC.A09* may regulate the expression of these six genes involved in the general phenylpropanoid pathway and lignin biosynthesis through *BnaTT8*.

### Disruption of *BnaTT8* or *BnaCCRL* confers lower SCC, lignin content, and higher SOC in *B. napus*

**Fig. 4:**
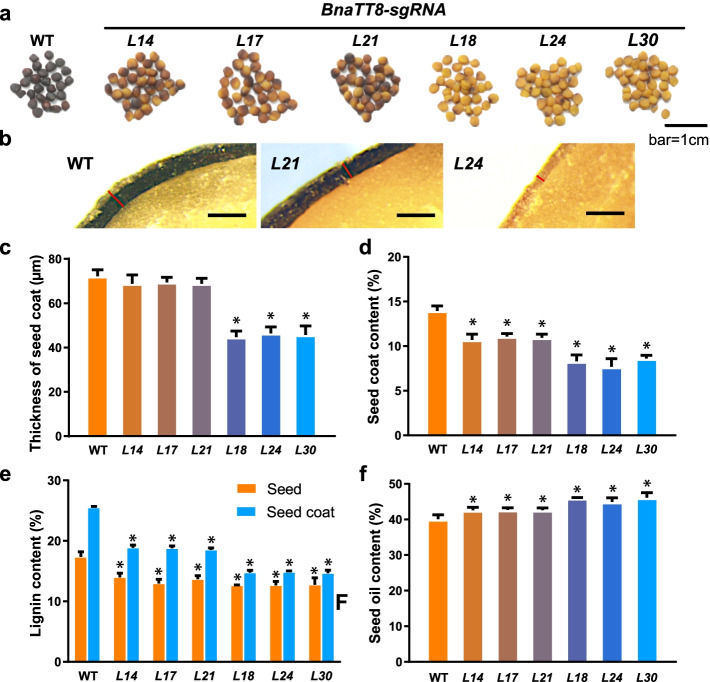
Functional characterization of *BnaTT8* as a positive regulator of seed lignin biosynthesis in *B. napus*. **a** Seeds of CRISPR/Cas9-induced *BnaA09.TT8* and *BnaC09.TT8* homozygous double mutant lines (*L14*, *L17*, *L21*, *L18*, *L24*, and *L30*). Bar = 1 cm. **b** the seed coats of *L21* and *L24*. Bar = 1 cm*.* Determination of thickness of seed coat (**c**), SCC (**d**), lignin content (**e**), and seed oil content (**f**) in the *BnaTT8* double knockout mutant seeds and the control (WT). Values are means ± SD of three biological replicates (*n* = 6). Student’s *t*-test was used for statistical analysis between the *BnaTT8*-sgRNA lines and WT (*, *P* < 0.05)

In order to clarify whether *BnaA09.TT8* and *BnaC09.TT8* affect SCC in *B. napus*, sgRNA1 and sgRNA2 targeting C-terminal conserved bHLH domain in the 7th exon were designed to generate *BnaA09.TT8* and *BnaC09.TT8* in *B. napus* cv. Westar using the CRISPR/Cas9 genome-editing system (Additional file 1: Fig. S6). A total of 43 T_0_-positive transgenic plants were generated and 12 T_2_ lines with homozygous mutations in both *BnaA09.TT8* and *BnaC09.TT8* identified by DNA sequencing of the PCR products of the target sites showed two different seed coat colors. Variegated seeded *BnaTT8-sgRNA* lines (*L14*, *L17*, and *L21*) and yellow seeded *BnaTT8-sgRNA* lines (*L18*, *L24*, and *L30*) were found to be *BnaTT8* double homozygote mutants (T_2_) which were chosen for further study (Fig. 4a). The data from the assessment of off-target effects of CRISPR/Cas9 showed no mutations at the potential off-target sites (Additional file 2: Table S18). According to the genotyping analysis (Additional file 1: Fig. S6), the detected homozygous mutations at the target sites caused frame shifts and premature termination, which resulted in a truncated bHLH conserve domain (29 amino acids) of *BnaA09.TT8* and a depletion of the whole bHLH conserve domain (43 amino acids) of *BnaC09.TT8* in *L14*, *L17*, and *L21* lines and non-functional *BnaA09.TT8* and *BnaC09.TT8* without the whole bHLH conserve domain in *L18*, *L24*, and *L30* lines (Additional file 1: Fig. S7). These results suggest that the N-terminal 29 aa of the bHLH conserve domain of *BnaA09.TT8* plays an important role in the *BnaTT8* function and these yellow seeded lines (*L18*, *L24* and *L30*) are *BnaTT8* double knockout mutants. Oxidized PAs content differs between the *BnaTT8* double knockout mutants and WT (Additional file 1: Fig. S13a-c).

In addition, the thickness of seed coat of the *BnaTT8* double knockout mutants (*L18*, *L24*, and *L30*) was reduced by 36.8% compared with that of WT, whereas no significant difference was found between the variegated seeded *BnaTT8-sgRNA* lines (*L14*, *L17*, and *L21*) and WT (Fig. 4b, c). It is worth noting that the SCC was reduced from 13.9% in WT to 8.29%, 7.68%, and 8.60% in the *BnaTT8* double knockout mutants (*L18*, *L24*, and *L30*) and to 10.7%, 11.1%, and 10.9% in the variegated seeded *BnaTT8-sgRNA* lines (*L14*, *L17*, and *L21*) (Fig. 4d). The lower SCC was attributed to the thinner seed coat of the *BnaTT8* double knockout mutants. The *BnaTT8* knockout mutants (*L18*, *L24*m and *L30*) had, on average, 27.0% to 42.2% reduced lignin content and the variegated seeded *BnaTT8-sgRNA* lines (*L14*, *L17* and *L21*) had, on average, 22.0% to 26.6% reduced lignin content in seed and seed coat compared with WT, respectively (Fig. 4e). Moreover, an approximately 4.90% to 6.03% increase in the SOC was found in the *BnaTT8* double knockout mutants (*L18*, *L24* and *L30*) and an approximately 2.50% to 2.63% increase in SOC was found in the variegated seeded *BnaTT8-sgRNA* lines (*L14*, *L17*, and *L21*) (Fig. 4f). The increase of SOC was found to be accompanied by an alteration in fatty acid composition in all the *BnaTT8-sgRNA* lines (Additional file 2: Table S19).

To further study whether the expression of lignin biosynthesis genes was impacted by *BnaTT8s* in *B. napus*, the expression levels of the genes involved in the general phenylpropanoid pathway and lignin biosynthesis pathway were quantitatively analyzed in developing seeds at 40 DAF. The expression of *4CL*, *hydroxycinnamoyl-CoA shikimate/quinate hydroxycinnamoyl transferase* (*HCT*), *ferulate 5-hydroxylase* (*F5H*), and *peroxidase* (*POD*) genes was not significantly altered by disruption of *BnaTT8* in *B. napus* (data not shown). The expression of *PAL* and *C4H* involved in the general phenylpropanoid pathway was down-regulated by 76.6% to 78.3% and 82.1% to 84.3% in the *BnaTT8* double knockout mutants compared with WT, respectively. The disruption of *BnaTT8* also significantly decreased the expression of *CCR*, *caffeoyl-CoA O-methyltransferase* (*CCoAOMT*), *caffeic acid O-methyltransferase* (*COMT*), and *cinnamyl alcohol dehydrogenase* (*CAD*) involved in the lignin biosynthesis pathway and *DFR* and *BAN* involved in the anthocyanin biosynthesis pathway compared with WT, suggesting that *BnaTT8s* may play a positive role in promoting the lignin biosynthesis as well as the anthocyanin biosynthesis through the general phenylpropanoid pathway (Additional file 1: Fig. S14).

**Fig. 5:**
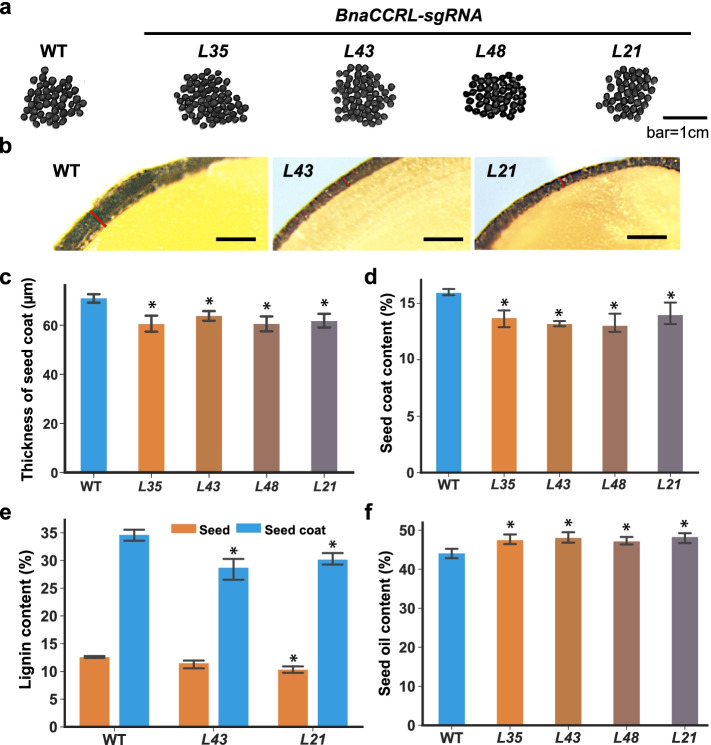
Functional characterization of *BnaCCRL* as a positive regulator of seed lignin biosynthesis in *B. napus*. **a** Seeds of CRISPR/Cas9-induced *BnaC07.CCRL* and *BnaA03.CCRL* homozygous double mutant lines (*L35*, *L43*, *L48*) and *BnaC07.CCRL*, *BnaA03.CCRL* and *BnaC05.CCRL* homozygous triple mutant line *L21*. Bar = 1 cm. **b** the seed coats of *L43* and *L21*. Bar = 1 cm*.* Determination of thickness of seed coat (**c**), SCC (**d**), lignin content (**e**), and seed oil content (**f**) in the *BnaCCRL* double or triple mutant seeds and the control (WT). Values are means ± SD of three biological replicates (*n* = 6). Student’s *t*-test was used for statistical analysis between the *BnaCCRL* lines and WT (*, *P* < 0.05)

*CCRL* gene has not been reported to be involved in seed coat formation in oilseeds. In Arabidopsis and *B. napus*. Although CCRL shares less than 40% protein sequence similarity with CCR (Additional file 1: Fig. S8) and is distant from BnaCCR1 and BnaCCR2 in phylogenetic tree (Additional file 1: Fig. S9), CCRL exhibited a reductase activity toward the p-coumaroyl-CoA, sinapoyl-CoA, or feruloyl-CoA like CCR (Additional file 1: Fig. S10). In order to study whether *BnaCCRL* affects SCC in *B. napus*, sgRNA1 and sgRNA2 targeting the conserved domain in the 3rd and 5th exon, respectively, were designed to generate *BnaCCRL* mutations in *B. napus* cv. Westar using the CRISPR/Cas9 genome-editing system (Additional file 1: Fig. S13). A total of 16 T_0_-positive transgenic plants were generated. Three T_2_ homozygous lines in which two homologs of *BnaCCRL*, *BnaC07.CCRL* and *BnaA03.CCRL* were mutated and one T_2_ homozygous lines in which three homologs of *BnaCCRL*, *BnaC07.CCRL*, *BnaA03.CCRL*, and *BnaC05.CCRL* were mutated (Fig. 5a; Additional file 1: Fig. S12, 11). The thickness of seed coat of *BnaCCRL-sgRNA-L21* and *BnaCCRL-sgRNA*-*L43* mutants was reduced by 10.1% to 13.1%, compared with that of WT (Fig. 5b, c). The SCC was reduced from 16.0% in WT to 13.0%-13.7% in the double mutants *BnaCCRL-sgRNA* lines (*L35*, *L43*, and *L48*) and 14.0% in the triple mutants *BnaCCRL-sgRNA-L21* (Fig. 5d). Additionally, the *BnaCCRL-sgRNA-L21* and *BnaCCRL-sgRNA*-*L43* mutants had, on average, 11.8% to 17.1% reduced lignin content in seed coat and the *BnaCCRL-sgRNA-L21* had, on average, 17.6% reduced lignin content in seed compared with WT, respectively (Fig. 5e). Moreover, an approximately 3.00% to 4.20% increase of SOC was found in the *BnaCCRL-sgRNA* lines (*L35*, *L43*, *L48*, and *L21*) with altered fatty acid composition (Fig. 5f; Additional file 2: Table S20).

Taken together, these results demonstrate that the *BnaTT8* or *BnaCCRL* mutation caused a significant reduction of SCC and increase of SOC. *BnaTT8* may modulate SCC by elevating the lignin content through the general phenylpropanoid pathway, whereas *BnaCCRL* may control SCC directly by lignin synthesis to thicken seed coat, leading to a negative effect on SOC in *B. napus*.

### Expression of TWAS significant gene showed opposite trends in SCC and SOC

**Fig. 6:**
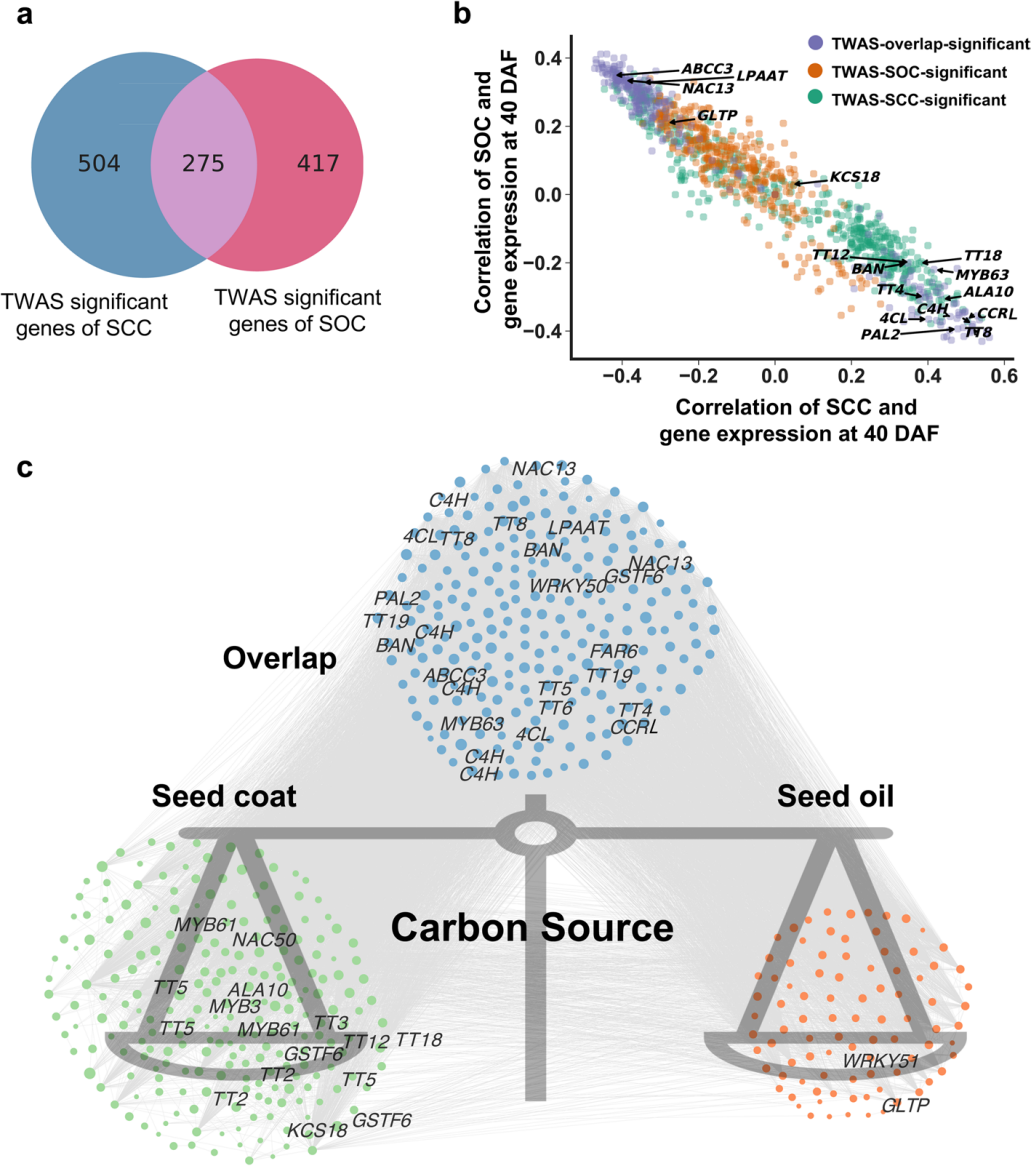
Correlation analysis between TWAS significant gene expression and SOC trait or SCC trait. **a** Venn diagram showing shared genes between significant genes identified by TWAS for SOC and SCC. **b** Correlation analysis between TWAS significant gene expression and SOC or SCC at 40 DAF. Each dot represents a gene. **c** Regulatory network of key genes for carbon source allocation in *B. napus*

Previous studies have shown that the SOC of *B. napus* showed a negative correlation with SCC [12, 27]. In this regard, we applied the 779 TWAS significant genes of SOC from previous studies [81] and the 692 TWAS significant genes of SCC obtained in this study to analyze the carbon source allocation between phenylpropanoid pathway and lipid accumulation (Fig. 6a; Additional file 2: Table S22). Among them, 275 TWAS significant genes were detected for both SCC trait and SOC trait (Fig. 6a; Additional file 2: Table S23). We further performed GO enrichment analysis (FDR<0.05) on these 275 overlapped significant genes. The results showed that these genes were mainly enriched in the metabolic pathways of oxidoreduction metabolism, transcription factor activity, flavonoid biosynthesis, and multiple amino acid transaminase activities (Additional file 1: Fig. S15). Subsequent correlation analysis of TWAS significant gene expression of SOC and SCC revealed the presence of many genes known to be associated with phenylpropanoid pathway and lipid metabolism whose expression showed opposite correlation with SOC and SCC. In particular, the degree of opposite correlation was more pronounced among some genes including *TT8*, *CCRL*, *C4H*, *PAL*, and *4CL* (Fig. 6b; Additional file 1: Fig. S16). To further investigate the key role of these genes in the carbon allocation of *B. napus*, we found that gene expression of *BnaC09.TT8* and *BnaC07.CCRL* were significantly positively correlated with SCC and negatively correlated with SOC, respectively (Additional file 1: Fig. S17-20).

To investigate the gene regulatory relationships during carbon source allocation in *B. napus*, we constructed a co-expression network using TWAS-SCC and TWAS-SOC significant genes (correlation > 0.6). Genes involved in the significant association between SOC and SCC were found to be closely linked, and genes affecting both SCC and SOC played a balancing role in the carbon source allocation process (Fig. 6c). These results suggest that overlapping regulatory networks for the synthesis of phenylpropanoid metabolites and fatty acids in the seed coat may exist. We have discovered a great number of genes involved in the carbon source allocation between phenylpropanoid pathway and oil synthesis pathway, which lay a basis for understanding that SCC reduction can contribute to increase of SOC by regulating the flow of carbon source into seed oil.

## Discussion

SCC, a quantitative trait formed during seed development, has been reported to be associated with seed color and lignin content in *B. napus* [11, 17, 22, 34, 35, 82]. Since the 1860s, yellow seed of oilseed rape with the low SCC and high SOC has attracted a great deal of attention from breeders. Numerous studies have revealed that SCC negatively correlates with the SOC [10–12, 36]. The present work revealed a great number of significant genes and genomic variations correlated with SCC using the genomic and transcriptomic data of developing seeds of a large natural *B. napus* population generated in our previous study [63]. The identified SCC-related QTLs and genes will help elucidate the regulatory mechanisms of the SCC trait in *B. napus*.

Different from GWAS, which identify SNPs and structural variations in the regions correlated with a phenotype, TWAS is a method to directly identify genes significantly associated with a phenotype due to the changes in gene expression levels. Our TWAS results revealed that there were 243 and 639 genes significantly associated with SCC at 20 DAF and 40 DAF, respectively (Fig. 1a, b). Most of the 103 overlapped genes between 20 DAF and 40 DAF were enriched in the secondary metabolite metabolic and biosynthetic pathway, especially in phenylpropane and flavonoid metabolic and biosynthetic pathway (Fig. 1c, d; Additional file 2: Table S5). Flavonoid and lignin biosynthesis are two branch pathways involved in phenylpropane biosynthesis pathway, sharing common precursors such as coumaroyl-CoA. PAs determined the seed coat color are synthesized by flavonoid pathway, involving the structural genes encoding catalytic enzymes such as TT4/CHS, TT5/CHI, TT6/F3H, TT7/F3'H, TT3/DFR, TT18/LDOX, BAN/ANR, and laccase (LAC) and the regulator genes encoding MYB, TFIIIA-like, WRKY-like, MADS-box-like, bHLH, MATE efflux, bZIP, C2H2, and HD-ZIP IV transcription factors [83–87]. In another branch pathway, the structural genes *HCT*, *C3H/C4H*, *CCR*, *F5H*, *COMT*, *CCoAOMT*, *CAD*, *LAC,* and *POD* are responsible for lignin biosynthesis [88, 89] that affects the seed coat thickness. Lots of flavonoid and lignin-related genes were identified from the TWAS results for the SCC trait (Fig. 1a). Among these genes, *BnaC07.CCRL* encoding a cinnamoyl-CoA-reductase-like protein is involved in lignin biosynthesis [78] and *BnaTT8s* encoding bHLH transcription factors are well-known regulators of flavonoid biosynthesis controlling seed coat color and seed fatty acid content in Arabidopsis and *B. napus* [80, 90]. According to the gene module analysis, the expression correlation analysis and the eGWAS results, *BnaC07.CCRL* and *BnaTT8s*, are predicted to be key regulators of SCC (Fig. 2a, b, e; Fig. 3a–c). The lower SCC was found in the seeds of *BnaTT8* knockout mutants and *BnaCCRL* knockout mutants generated by CRISPR/Cas9 gene editing technology, respectively (Fig. 4d; Fig. 5d), suggesting the accuracy of TWAS and module analyses for SCC trait.

More interestingly, *qSCC.A09* identified by GWAS were found to function upstream of *BnaC07.CCRL* and *BnaTT8s* using eGWAS. Moreover, to further study how *BnaTT8* affects SCC, eGWAS was performed using *BnaTT8* as a covariant and differentially expressed genes in the *BnaTT8* mutant [80] were also analyzed for identification of the downstream genes. Thus, six genes including *BnaC07.CCRL* and some homologs of *PAL* and *C4H* were predicted to be regulated by *BnaTT8* (Fig. 3d). Gene expression of *PAL* and *C4H* involved in the general phenylpropanoid pathway was significantly downregulated when *BnaTT8* genes were knockout (Additional file 1: Fig. S14), consistent with the eGWAS results (Fig. 3d). Additionally, it was found that not only the expression levels of *DFR* and *BAN* involved in flavonoid biosynthesis pathway but also that of *CCoAOMT*, *CCR*, *COMT*, and *CAD* involved in lignin biosynthesis pathway were downregulated in the *BnaTT8* knockout mutants (Additional file 1: Fig. S14), suggesting *BnaTT8s* are also an important regulator of the lignin biosynthesis, as well as flavonoid biosynthesis. Although *BnaTT8s* shared a similar expression pattern with the lignin biosynthetic genes, no TT8 binding motif was found in the promoter region of these lignin synthesis genes. Taken together, our results indicate that BnaTT8 is a master regulator of SCC, which may indirectly regulate lignin content through the general phenylpropane pathway when flavonoid was synthesized by the direct regulation of *BnaTT8* in *B. napus*.

Based on GWAS for SCC trait, we identified three QTLs on chromosomes A09 and C05, including *qSCC.C05.1*, *qSCC.C05.2*, and *qSCC.A09* (Fig. 3a; Additional file 2: Table S11). Moreover, *qSCC.A09* was co-located with the eQTL results from eGWAS for *BnaC07.CCRL*, *BnaTT8s*, and some other TWAS-significant genes (Fig. 3b, c; Additional file 2: Table S13), suggesting *qSCC.A09* is a regulatory hotspot of SCC. Interestingly, all these three QTLs have also been mapped by GWAS for SOC trait or lignin content by previous studies [12, 27, 34], suggesting a strong correlation among SCC, SOC, and lignin content. In addition, M65, M139, M97, and M79 significantly associated with SCC at different stages of the seed development were generated by the gene module analysis of TWAS results (Fig. 2a–d), among them M65, M139, and M79 have also been identified to be associated with SOC trait [63]. These results indicate that there is a close correlation between seed coat formation and seed oil accumulation, showing an overlapped gene expression regulatory network.

The MBW complex formed by TRANSPARENT TESTA 2 (TT2), TT8, and TRANSPARENT TESTA GLABRA 1 (TTG1) is one of the most important regulatory complex for flavonoid biosynthesis, which can directly activate the expression of BAN (BANYULS) and DFR (Dihydroflavonol-4-reductase) and control anthocyanin and proanthocyanidins synthesis. *BnaTT2* or *BnaTT8* mutation can disrupt the function of the ternary complex, leading to yellow seed coats and high SOC [80, 91]. However, it remains unclear that how BnaTT2 or BnaTT8 regulate the SOC. The editing sites of *BnaTT8* genes in this study (Additional file 1: Fig. S6) are different from that in the previous study [80]. Due to the different editing sites, we obtained the *BnaTT8* mutants with different seed coat colors, including variegated seeded *BnaTT8-sgRNA* lines (L14, L17, and L21) and yellow seeded *BnaTT8-sgRNA* lines (L18, L24, and L30) (Fig. 4a). Our results revealed that BnaTT8s can affect SOC through regulation of SCC.

Moreover, we also found that the expression of significantly associated genes identified by TWAS, including *TT8*, *CCRL*, *C4H*, *PAL*, *4CL*, *LPAAT*, *GLTP*, and many other genes related to phenylpropane metabolism and lipid accumulation, showed an opposite correlation between SCC and SOC (Fig. 6b; Additional file 1: Fig. S16). The *BnaTT8* knockout double mutants generated here also showed a typical yellow seeded traits with the high SOC (Fig. 4a, f), which was consistent with the results [80]. We also found that the disruption of *BnaCCRL* also caused the significantly increased SOC (Fig. 5f). Our results indicate *BnaTT8* and *BnaCCRL* may indirectly affect SOC by positive regulation of SCC through promoting the lignin biosynthesis. From the viewpoint of secondary metabolism, when the general phenylpropanoid pathway was turned down, more carbon source, malonyl-CoA, will flow into oil biosynthesis [92]. Under the condition of fixed seed size and limited carbon source, the thickening of the secondary wall affected by lignin synthesis and the accumulation of flavonoids in the seed coat not only compress the specific proportion of embryo, but also compete with the biosynthesis of fatty acids for the limited carbon source in the seed, thereby affecting seed oil accumulation.

## Conclusions

Taken together, this study provides insights into the dissection of the genetic basis of SCC by TWAS and GWAS in *B. napus*. Over seven hundred genes, four gene modules, and three QTLs were identified to be significantly associated with SCC from the multi-omics data. It was revealed that some significantly associated genes identified by TWAS for SCC and SOC traits may play a key role in the allocation of carbon sources in *B. napus* seeds. The identified SCC-related candidate genes and loci will promote the studies on the mechanism of seed coat formation and provide valuable information for the breeding of *B. napus* with low SCC and high SOC.

## Methods

### Plant materials and traits measurements

A total of 382 *B. napus* accessions re-sequenced in previous studies [63] were collected from spring, winter, and semi-winter accessions and cultivated under natural conditions on the experimental farm of the Huazhong Agricultural University, Wuhan, China in 2016–2017. The T_0_ and T_1_ transgenic and WT plants were grown in a greenhouse (16/8 h of light/dark at 22 °C) in 2016 and 2017, respectively. The three selected homozygous *BnaTT8* knockout mutants (T_2_) without T-DNA were grown on the experimental farm of Huazhong Agriculture University, Wuhan, China in 2018~2019. All the field experiments followed a randomized complete block design with three replications. The seed coat was separated from the embryo with tweezers under a microscope. The seed weight and the seed coat weight were weighed with an electronic balance of ten thousandths. The SCC was calculated as the ratio of the seed coat weight to the seed weight. The oil content and protein content of dried seeds were determined by a Foss NIR Systems 5000 near-infrared reflectance spectroscope using the parameters described by Gan et al. [93]. The FA composition in mature seeds was determined using gas chromatography (GC) system with a Model 6890 GC analyzer (Agilent Technologies, Inc., Wilmington, DE) as described previously [94]. Yield-related traits, including plant height, length of main inflorescence branch, number of siliques on the main inflorescence, number of first branch, branch height, number of siliques per plant, length per silique, number of seeds per silique, 1000-seed weight, and seed yield per plant, were measured as described previously [95].

### Variation calling, genotype filling, and annotation

We applied the results of the genotype imputation of the previous article [63]. The genome sequences of *B. napus* were downloaded from GENOSCOPE (http://www.genoscope.cns.fr/brassicanapus/) [96]. BWA [97] software was used to align the reads to the reference genome and the PCR duplicates were removed with SAMTools [98]. GATK v3.6 [99] was used to identify the existed sequencing variations in the 382 varieties and merge the files. SNPs and InDels with lower mapping quality (MQ <20) or reads with lower sequencing depth (DP < 50) were filtered. After obtaining the genotype, the missing genotype was estimated by the LD-KNN algorithm [100].

### Genome-wide association studies and expression genome-wide association study

A total of 8,593,156 SNPs (MAF > 0.05) were used for genome-wide association studies (GWAS) in the whole population. The significance threshold of association was calculated by Genetic Type I error calculator (GEC) software [101], the calculated significance threshold is 7.96×10^−6^. We used the GEMMA [102] software package to perform correlation analysis on multiple traits in Wuhan using a mixed linear model [103]. eGWAS was performed using gene expression as phenotypic data for GWAS [54].

### Transcriptome-wide association Studies, GO enrichment analysis, and independent component analysis (ICA) for expression data

The transcriptome data and the ICA gene modules were obtained from previous studies [63], and we filtered out the genes according to the 95th percentile of log_2_-transformed expression values, and low-expression genes in the population were removed in subsequent analyses. Linear analysis and mixed linear model of EMMAX [104] were used for correlation analysis. FDR corrected *p* value ≤ 0.05 was used as the significance threshold. Proteins in *B. napus* were aligned to all Arabidopsis proteins by BLASTP [105], and the most significant proteins in Arabidopsis based on a probability threshold of 10^−5^ were selected for enrichment analysis.

### Construction of the CRISPR/Cas9 vector and plant transformation

The CRISPR/Cas9 genome-editing system was utilized for gene editing of *BnaTT8* and *BnaCCRL* in this study, respectively [106]. The primer sequences for the sgRNA vector construction were listed in (Additional file 2: Table S21). Agrobacterium tumefaciens-mediated hypocotyl transformation in *B. napus* was conducted as previously described [107]. The *B. napus* cv. Westar was used as the transformation receptor.

### Quantitative RT-PCR

To analyze the expression level of the genes involved in phenylpropane metabolic and lignin biosynthesis pathway, quantitative RT-PCR was performed. Total RNA was isolated from the developing seeds at 4 weeks after flowering using the TRIzol® Reagent (Invitrogen). RNA samples were reverse-transcribed using M-MLV reverse transcriptase (Promega) and oligo (dT) 15 primer, according to the manufacturer’s instructions. The expression level of the *B. napus* β-actin gene (AF111812) was used as a loading control. The primer sequences for quantitative RT-PCR analyses were listed in (Additional file 2: Table S21).

### Total lignin assay

Seed coats and seeds were collected from the homozygous *BnaTT8-sgRNA* lines (*L14*, *L17*, *L21*, *L18*, *L24*, and *L30*), the homozygous *BnaCCRL-sgRNA* lines (L43 and L21), and the *B. napus* cv. Westar (WT), respectively. Total lignin content was measured in the seed coats and whole seeds using a two-step acid hydrolysis method as previously described [108]. Acid-insoluble and acid-soluble are two types of lignin. The acid-insoluble lignin (AIL) was calculated gravimetrically after correction for ash, and the acid-soluble lignin (ASL) was measured by UV spectroscopy. The following formula was used: for acid-insoluble lignin determination, the 0.5-g sample was recorded as W1. The weight of the crucible and dry residue was recorded to the nearest 0.1 mg (W2). The weight of the crucibles and ash were recorded to the nearest 0.1 mg (W3). All experiments were carried out in triplicate.

$$\mathrm{AIL}\ \left(\%\right)=\left(\mathrm{W}2+\mathrm{W}3\right)\times 100/\mathrm{W}1\%.$$

For acid-insoluble lignin determination, where *A* is the absorption value, *D* is the dilution ratio of the sample, and *K* (the absorptivity constant) = 110 L/g/cm.

$$\mathrm{ASL}\ \left(\%\right)=\left(\mathrm{A}\times \mathrm{D}\times \mathrm{V}/1000\times \mathrm{K}\times \mathrm{W}1\right)\ 100\%.$$

Total lignin content (%) = AIL (%) + ASL (%)

### Vanillin and DMACA staining and quantification of insoluble PCs

Flavan-3,4-diols and flavan-4-ols as monomers of PAs in detached developing seed coats were detected by vanillin. The seed coats were incubated in a solution of 0.5% (w/v) vanillin in 5 N HCl for 15 min at room temperature [109]. PAs and flavon-3-ols in detached developing seed embryos were detected by DMACA. The seed embryos were stained with 2% DMACA dissolved in a 6 N HCl/95% ethanol mixture (1:1, v/v) for 30 min at room temperature and then were washed for 15 min with water [110]. Insoluble procyanidin oligomers were measured using the hot butanol-HCl method described by Auger et al. [111]. 50 entire seeds were ground with 9 mL acetone/water/TFA mixture (80:20:0.05, v/v/v) for 5 min and then sonicated for 15 min at 4 °C. The pellets were collected after centrifugation (18,000*g*, 10 min at 4 °C). After added approximately 15 mL of butanol-HCl (95:5, v/v) and 500 μL of 2% ferricammonium sulfate (w/v) in 2 N HCl, the pellets were heated at 95 °C for 3 h. Absorbance of pink-red cyanidin released after cleavage of PAs was recorded at a wavelength of 550 nm.

### Protein purification and enzymatic assay of BnaCCRL

The full-length cDNA of BnaCCRL was cloned and inserted into pET-28b-Sumo vector at the BamHI/XhoI cloning sites using ClonExpress® II One Step Cloning Kit. The primer sequences used for cloning were listed in (Additional file 2: Table S21). pET-28b-Sumo-BnaCCRL was then transformed into the *E.coli* BL21 (DE3) cells. Protein expression was induced by addition of 0.2 mM IPTG (isopropyl-b-D-thiogalactoside) at 16 °C. The pallets were collected by centrifugation at 4000 rpm for 20 min. Cells were resuspended in lysis buffer (150 mM NaCl, 25 mM Tris-HCl pH8.0) and lysed by a cell homogenizer. The lysate was centrifuged at 14,000 rpm for 1 h to remove cellular debris. The supernatant was loaded onto a Ni-NTA superflow affinity column, and the resin was washed by wash buffer (150 mM NaCl, 25 mM Tris-HCl pH8.0). The protein was eluted in fractions containing 25 mM Tris-HCl pH8.0 and 300 mM imidazole. The concentration of purified protein was measured with a protein assay kit (Bio-Rad). The purified proteins were separated by 12% SDS-PAGE, stained with Coomassie Brilliant Blue, and immunoblotted using His-tag antibodies (1:3000 dilution; A-5588, Sigma).

The activities of BnaCCRL were analyzed by the reduction of p-coumaroyl-CoA, sinapoyl-CoA, or feruloyl-CoA in the presence of NADPH based on the functions in the biosynthesis pathway. Total 500 μl standard mixture for CCR activity analysis contains 82 μM substrates, 160 μM NADPH, and 100 mM phosphate buffer at pH6.2 with 15 μg purified proteins. The reaction was underdone in 500 μl standard with 82 μM p-coumaroyl-CoA, sinapoyl-CoA, or feruloyl-CoA, respectively. Absorbance was recorded at a wavelength of 366 nm [112]. After the reaction was initiated, the absorption values were monitored every minute. Enzymatic activity was calculated using molar absorption coefficients of substrates and products at 366 nm provided by Stöekigt and Zenk [113].

## Supplementary Information

**Additional file 1:** **Fig. S1** to **Fig. S20**.

**Additional file 2:** **Table S1** to **Table S23**.

**Additional file 3.** Review history.

## Data Availability

Genome sequencing and RNA-seq data were generated in our previous work (62). Data generated in this study are submitted as Additional files with this manuscript. Other data are also available from the corresponding authors upon request.
