# Supplementary material for: Multi-omics analysis dissects the genetic architecture of seed coat content in Brassica napus
Source: Genome Biol. 2022 Mar 28;23:86. doi: 10.1186/s13059-022-02647-5 (PMC8962237; doi:10.1186/s13059-022-02647-5)
Supplement: Supplementary file 1 — Additional file 1: Fig. S1 to Fig. S20. [file 13059_2022_2647_MOESM1_ESM.pdf]

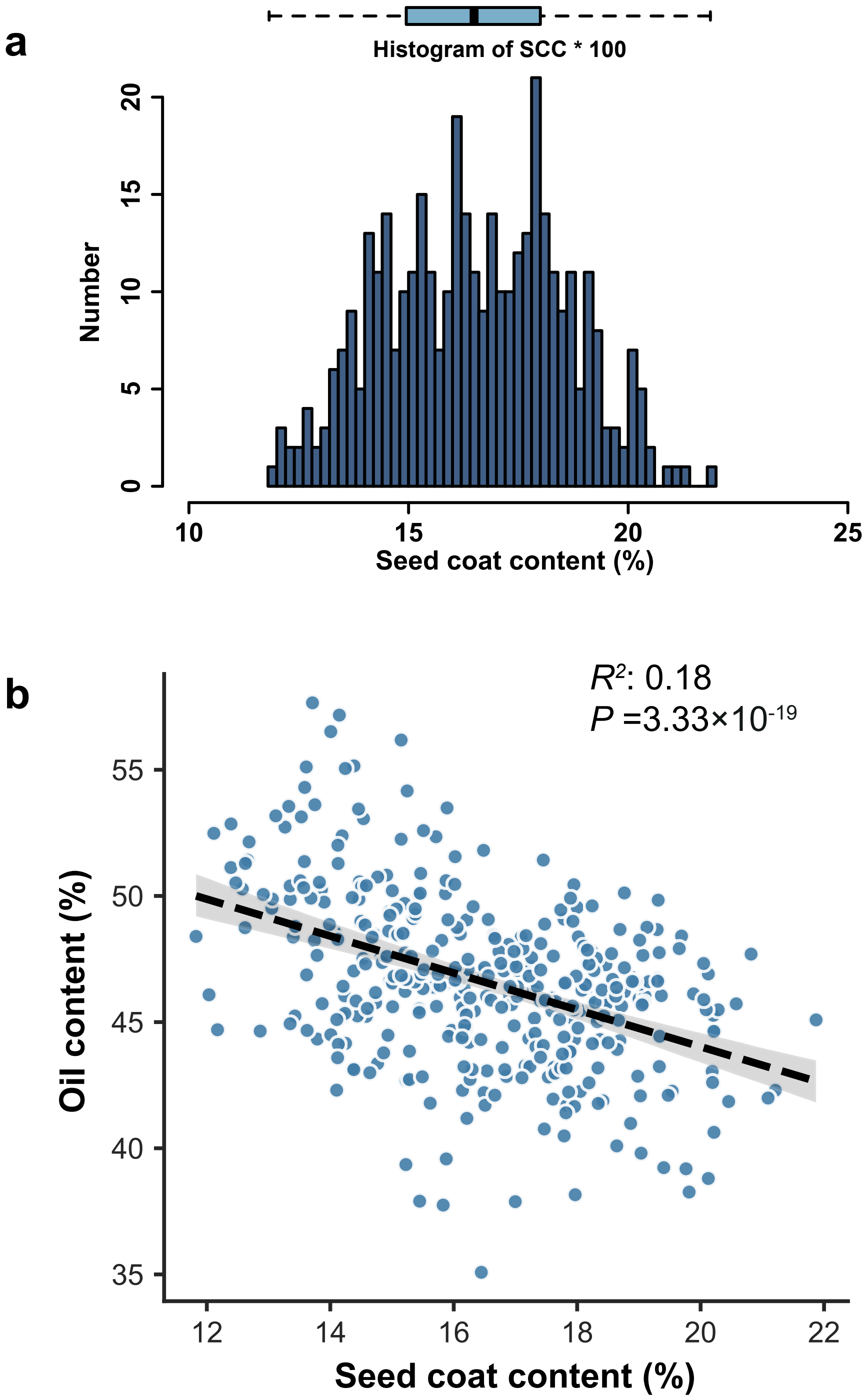

Fig. S1 (a) Phenotypic distribution for SCC. (b) Correlation analysis between SOC and SCC trait.

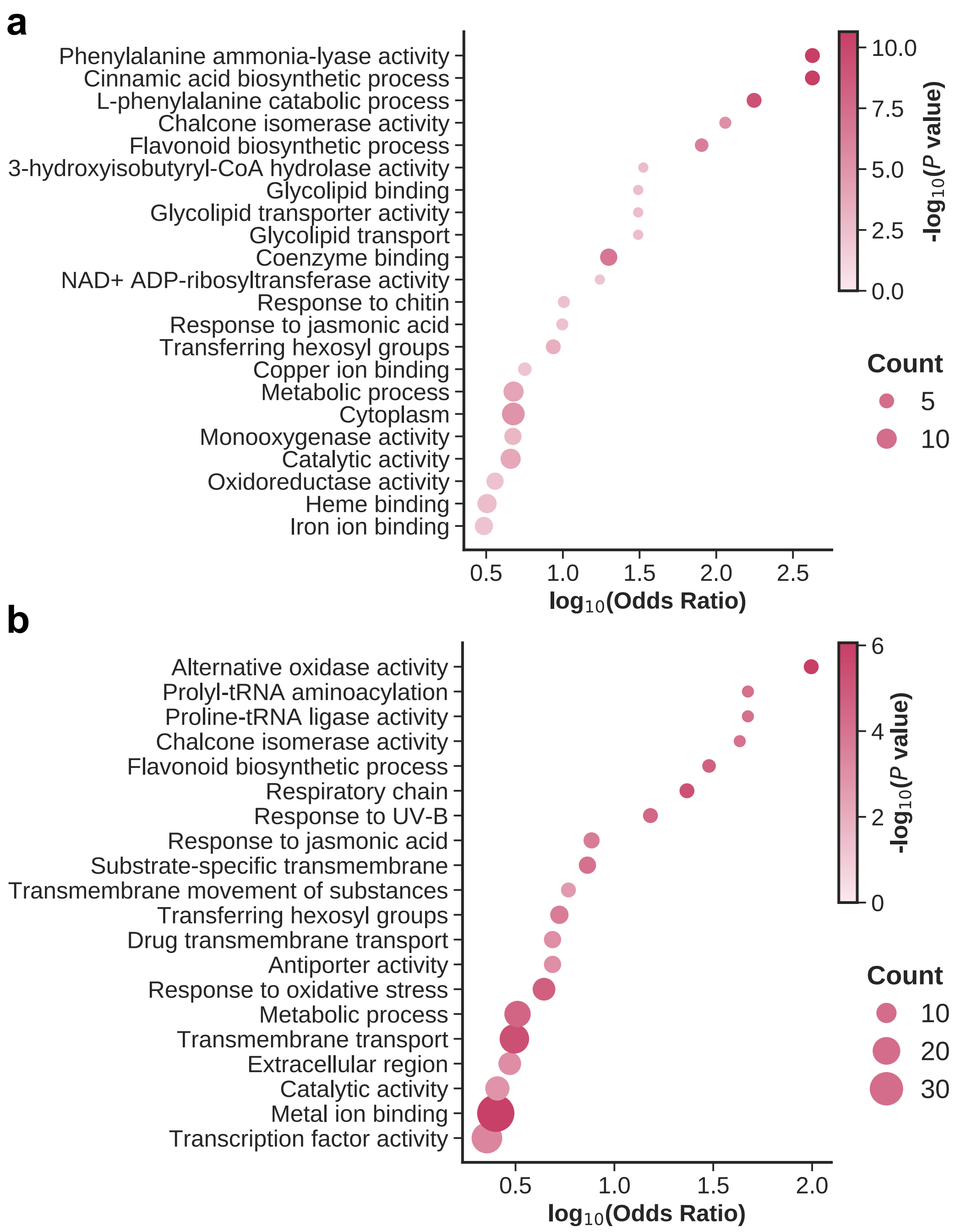

Fig. S2 (a) GO enrichment of TWAS significant genes for SCC at 20DAF. (b) GO enrichment of TWAS significant genes for SCC at 40DAF.

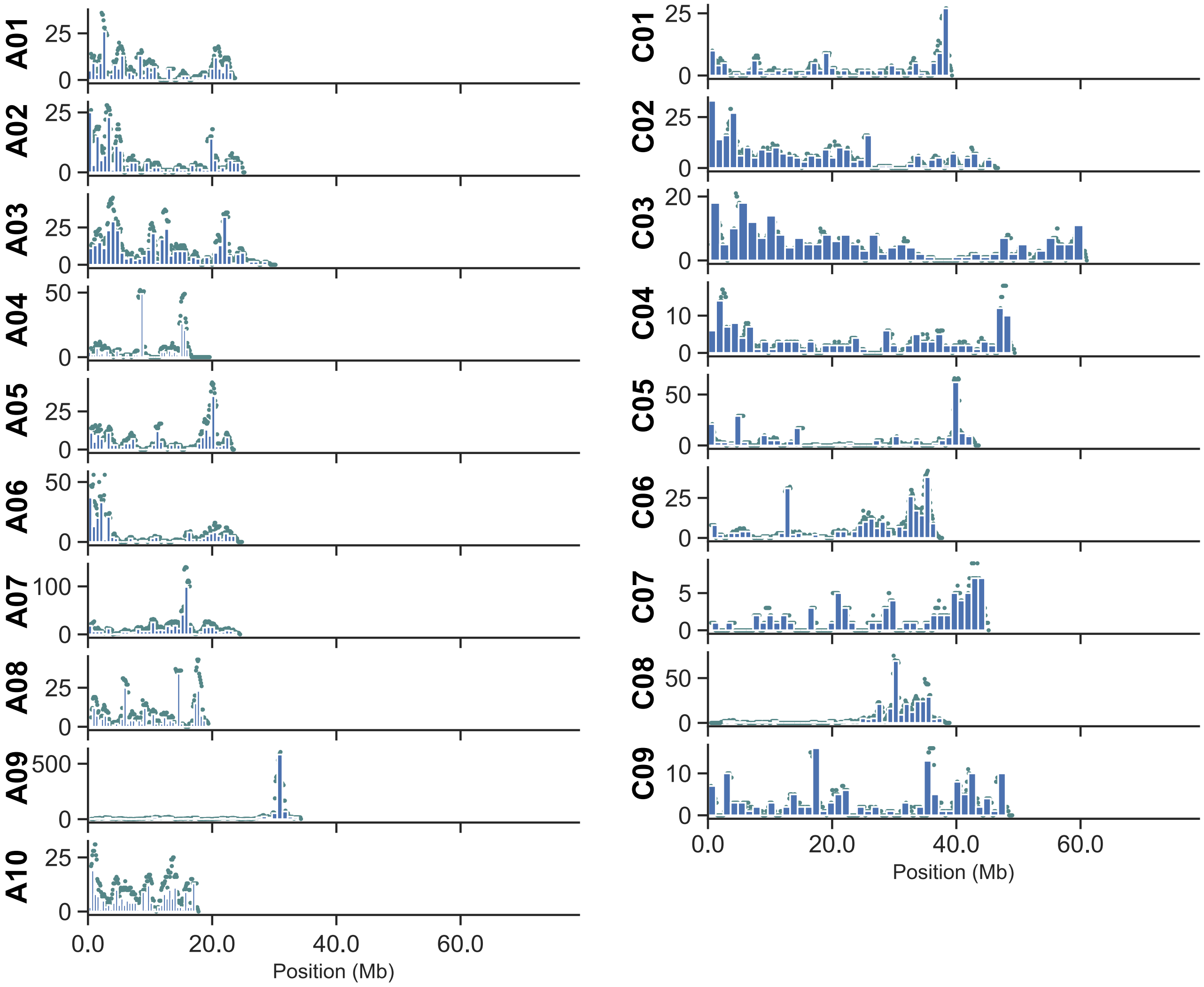

Fig. S3 The density plot and histogram of eQTL location for TWAS-SOC significant genes at 40 DAF.

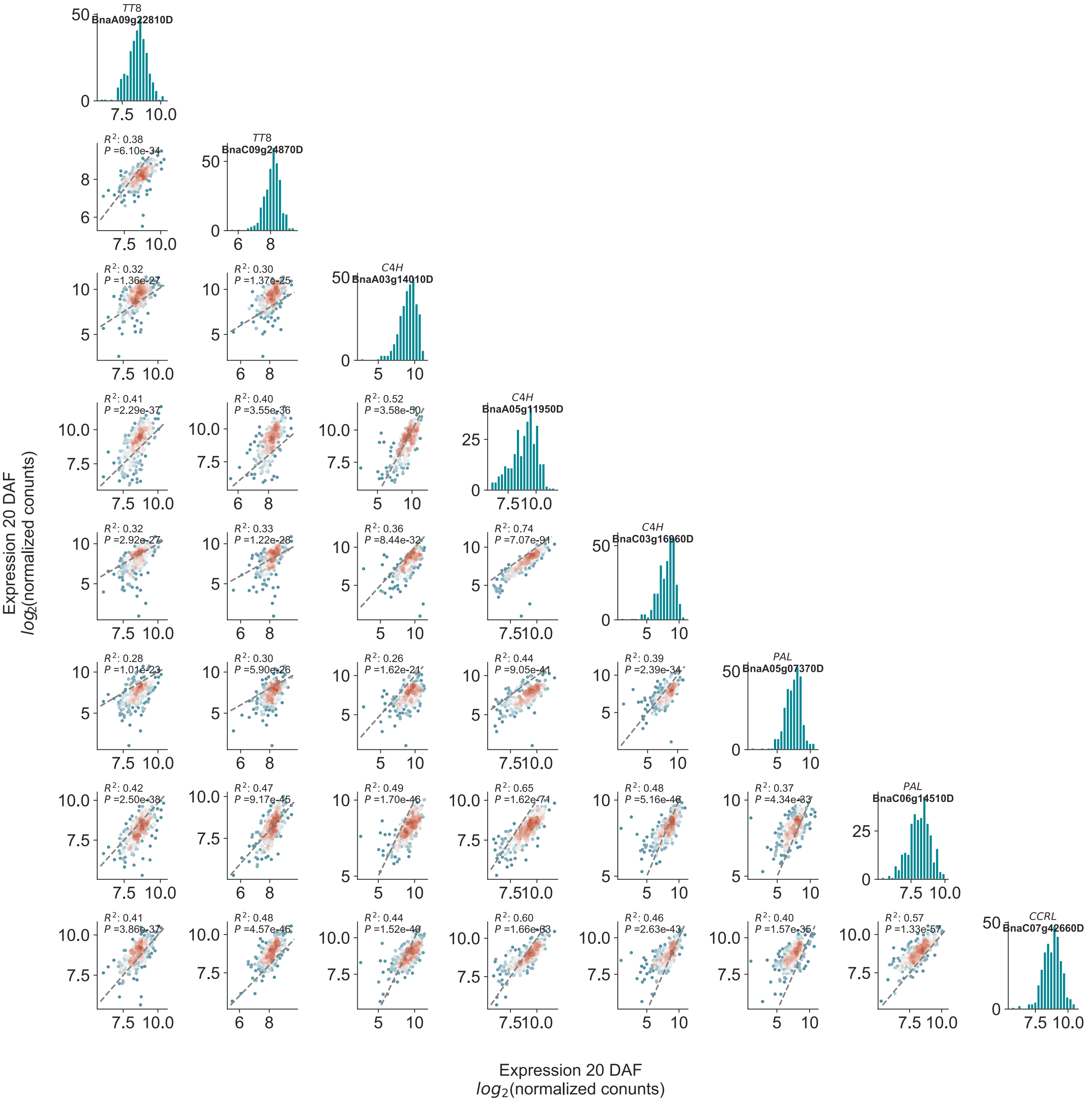

Fig. S4 Histogram of gene expression distribution and correlation coefficient between *BnaTT8s* and the expression of *PAL, C4H* and *CCRL* in 257 accessions at 20 DAF. R^2 is the square of Pearson correlation coefficient and the *P*-value is used for testing non-correlation.

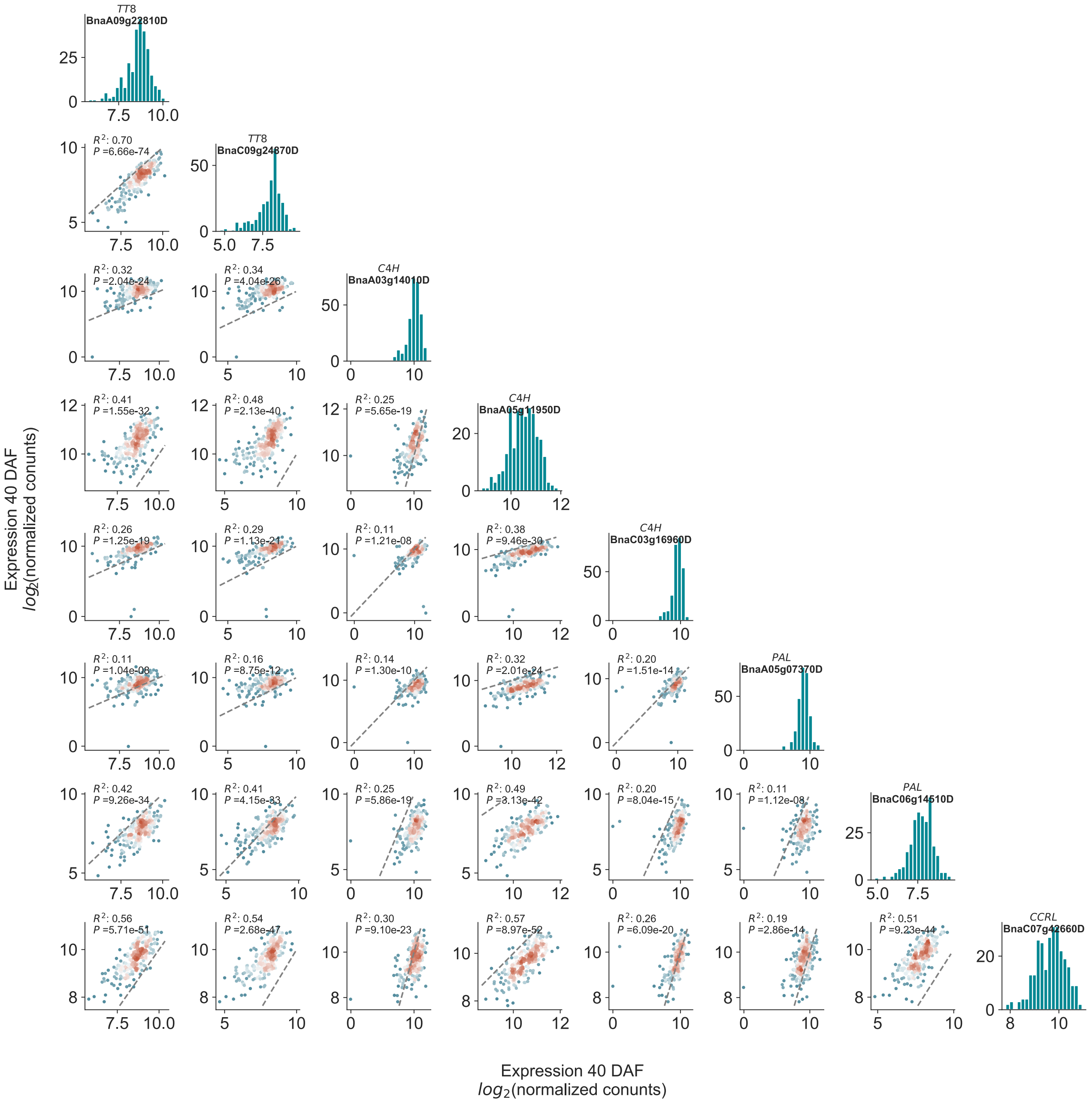

Fig. S5 Histogram of gene expression distribution and correlation coefficient between *BnaTT8s* and the expression of *PAL, C4H* and *CCRL* in 257 accessions at 40 DAF. R^2 is the square of Pearson correlation coefficient and the *P*-value is used for testing non-correlation.

Bna.A9-sgRNA1

```
L14:ATGGACATGATGAATCTAA-TGGAGGAAGGCGGA     (WT)
L17:ATGGACATGATGAATCTAA-TGGAGGAAGGCGGA     (WT)
L21:ATGGACATGATGAATCTAA-TGGAGGAAGGCGGA     (WT)
L18:ATGGACATGATGAATCTAAATGGAGGAAGGCGGA     (insertion)
L24:ATGGACATGATGAATCTAA----AGGAAGG-GGA     (deletion)
L30:ATGGACATGATGAATCTAA--G-AGGAAGGCGGA     (deletion)
Ref:ATGGACATGATGAATCTAA-TGGAGGAAGGCGGA
```

Bna.A9-sgRNA2

```
L14:TTGGTTCCATTTGTGA-----GATGGATAAAGTC     (deletion)
L17:TTGGTTCCATTTGTGA-----GATGGATAAAGTC     (deletion)
L21:TTGGTTCCATTTGTGA-----GATGGATAAAGTC     (deletion)
L18:TTGGTTCCATTTG---------ATGGATAAAGTC     (deletion)
L24:TTGGTTCCATTTGTGACC---GATGGATAAAGTC     (deletion)
L30:TTGGTTCCATTTGTGAC-AAAGA--GATAAAGTC     (deletion and insertion)
Ref:TTGGTTCCATTTGTGACCAA-GATGGATAAAGTC
```

Bna.C9-sgRNA1

```
L14:ATGGACATGATGAATCTAATTGGAGGATAGCTAGCTA  (insertion)
L17:ATGGACATGATGAATCTAA--GGAGGATAGCTAGCTA  (deletion)
L21:ATGGACATGATGAATCTA-------GGTAGCTAGCTA  (deletion)
L18:ATGGACATGATGAATCTAATTGGAGGATAGCTAGCTA  (insertion)
L24:ATGGACATGATGAATCTAATTGGAGGATAGCTAGCTA  (insertion)
L30:ATGGACATGATGAATTCTAATGGAGGATAGCTAGCTA  (insertion)
Ref:ATGGACATGATGAATCTAA-TGGAGGATAGCTAGCTA
```

Bna.C9-sgRNA2

```
L14:TTGGTTCCATTTGTGA----AGATGGATAAAGTCTCGA (deletion and insertion)
L17:TTGGTTCCATTTGTGA-----GATGGATAAAGTCTCGA (deletion)
L21:TTGGTTCCATTTGTGACCAAAGATGGATAAAGTCTCGA (insertion)
L18:TTGGTTCCATTTGTGACCAA-GATGGATAAAGTCTCGA (WT)
L24:TTGGTACATGTAGAGAGC---------------CTCGA (deletion and insertion)
L30:TTGGTAGATGGAGAG----------------------A (deletion and insertion)
Ref:TTCAGTGCACGTGTGACCAA-GATGGGCATACGCTCGA
```

Fig. S6 Genotype of *BnaTT8-sgRNA* mutant lines in the T_0 generation. Nucleotides are shown with sgRNA target (red), PAM sequence (green) and mutation sites (blue) indicated.

BnaA09.TT8

```
L14: FLHDNTKNKRLPREELNHVVAERRRREKLNERFITLRSLVPFVRWIKSRS 400
L17: FLHDNTKNKRLPREELNHVVAERRRREKLNERFITLRSLVPFVRWIKSRS 400
L21: FLHDNTKNKRLPREELNHVVAERRRREKLNERFITLRSLVPFVRWIKSRS 400
Ref: FLHDNTKNKRLPREELNHVVAERRRREKLNERFITLRSLVPFVTKMDKVS 400

L14: LETPLNT* 407
L17: LETPLNT* 407
L21: LETPLNT* 407
Ref: ILGDTIEYVNHLSKRIHELESTHHEPNQKRMRIGKGRTWEEVEVSIIESD 450
```

```
L18: SNQNLLSDFHIEATNSLDTHMDMMNLNGGRRKLFSDSINTSHVTTHKSSF 300
L24: SNQNLLSDFHIEATNSLDTHMDMMNLKEGEIILRQYQHFSCHNPQVFFQI 300
L30: SNQNLLSDFHIEATNSLDTHMDMMNLRGRRKLFSDSINTSHVTTHKSSFR 300
Ref: SNQNLLSDFHIEATNSLDTHMDMMNLMEEGGNYSQTVSTLLMSQPTSLLS 300

L18: RFSFHIFLRSIIVCLVES* 318
L24: QFPHLLTFNHRLSRGELRMSKSISNISEWRKRRGHRRNGCSNT* 343
L30: FSFHIFLRSIIVCLVES* 317
Ref: DSVSTSSYVQSSFVSWRVENVKEHQQYQRVEKAAWSSSQWMLKHIILKVP 350
Ref: FLHDNTKNKRLPREELNHVVAERRRREKLNERFITLRSLVPFVTKMDKVS 400
Ref: ILGDTIEYVNHLSKRIHELESTHHEPNQKRMRIGKGRTWEEVEVSIIESD 450
```

BnaC09.TT8

```
L14: SNQNLLSDFHIEATNSLDTHMDMMNLIGGRRKLFSDSINTSHVTTHQSSF 300
L17: SNQNLLSDFHIEATNSLDTHMDMMNLRRKAEIILRQYQHFSCHNPPVFFQ 300
L21: SNQNLLSDFHIEATNSLDTHMDMMNLGRRKLFSDSINTSHVTTHQSSFRF 300
L18: SNQNLLSDFHIEATNSLDTHMDMMNLIGGRRKLFSDSINTSHVTTHQSSF 300
L24: SNQNLLSDFHIEATNSLDTHMDMMNLIGGRRKLFSDSINTSHVTTHQSSF 300
L30: SNQNLLSDFHIEATNSLDTHMDMMNSNGGRRKLFSDSINTSHVTTHQSSF 300
Ref: SNQNLLSDFHIEATNSLDTHMDMMNLMEEGGNYSQTVSTLLMSQPTSLLS 300

L14: RFSFHIFLRSIIVYIVES* 318
L17: IQFPHLLTFNHRLYRGELRMSKSISNISEWKKRRLRRRNGCSNT* 344
L21: SFHIFLRSIIVYIVES* 316
L18: RFSFHIFLRSIIVYIVES* 318
L24: RFSFHIFLRSIIVYIVES* 318
L30: RFSFHIFLRSIIVYIVES* 318
Ref: DSVSTSSYVQSSFISWRVENVKEHQQYQRVEKAASSSSQWMLKHIILKVP 350
Ref: FLHDNTKNKRLPREELNHVVAERRRREKLNERFITLRSLVPFVTKMDKVS 400
Ref: ILGDTIEYVNHLSKRIHELESTHHEPNQKRMRIGKGRTWEEVEVSIIESD 450
```

Fig. S7 Amino acid sequence alignment of the target sites derived from WT and *BnaTT8-sgRNA* lines.

```
BnaA07g20090D_CCR2  ............................................................
BnaC06g19540D_CCR2  ............................................................
BnaA02g36250D_CCR2  .....MPADGKLVCVTGAGGYIASWIVKLLLERGYTVRGTVR.NPADPKNNHLRELQGAK
BnaC02g46610D_CCR2  .....MPADGKLVCVTGAGGYIASWIVKLLLERGYTVRGTVR.NPADPKNNHLRELQGAK
BnaA07g35280D_CCR2  .....MPADGKLVCVTGAGGYIASWIVKLLLERGYTVRGTVR.NPADPKNNHLRELDGAK
BnaC06g40190D_CCR2  .....MPADGKLVCVTGAGGYIASWIVKLLLERGYTVRGTVR.NPADPKNNHLRELDGAK
BnaA09g56490D_CCR1  MPVDESTPVGKTVCVTGAGGYIASWIVKLLLERGYTVKGTVR.NPDDPKNTHLRELEGAK
BnaC08g38580D_CCR1  MPVDESTAVGKTVCVTGAGGYIASWIVKLLLERGYTVKGTVR.NPDDPKNTHLRELEGAK
BnaC05g12180D_CCR1  MPVDESSPAGKTVCVTGAGGYIASWIVKLLLERGYTVKGTVR.NPDDPKNTHLRELEGAK
BnaA06g10620D_CCR1  MPLAESSPAGKTVCVTGAGGYIASWIVKLLLERGYTVKGTVR.NPDDPKNTHLRELEGAK
BnaC07g42660D_CCRL  ...MDQEKSHSCCCVLDASTYVGFWILKKLLSRGYSVHAAIRKNGDSEIEETIREMEATE

BnaA07g20090D_CCR2  ............................................................
BnaC06g19540D_CCR2  ............................................................
BnaA02g36250D_CCR2  ERLTLHSADLLDYEALCATIDGCDGVFHTASPMTDDPETMLEPAVNGAKFVIDAAAKAK.
BnaC02g46610D_CCR2  ERLTLHSADLLDYEALSATIDGCDGVFHTASPMTDDPETMLEPAVNGAKFVIDAAAKAK.
BnaA07g35280D_CCR2  ERLTLHSADLLDYEALCAAIDGCDGVFHTASPMTDDPETMLEPAVNGAKFVIDAAAKAK.
BnaC06g40190D_CCR2  ERLTLHSADLLDYEALCAAIDGCDGVFHTASPMTDDPETMLEPAVNGAKFVIDAAAKAE.
BnaA09g56490D_CCR1  ERLILCKADLQDYEALKAAIDGCDGVFHTASPVTDDPEQMVEPAVNGAKFVINAAAEAK.
BnaC08g38580D_CCR1  ERLILCKADLQDYDALKSAIDGCDGVFHTASPVTDDPEQMVEPAVNGAKFVINAAAEAK.
BnaC05g12180D_CCR1  ERLILCKADLQDYEALKAAIDGCDGVFHTASPVTDDPEQMVEPAVNGAKFVINAAAEAK.
BnaA06g10620D_CCR1  ERLILCKADLQDYEALKTAIDGCDGVFHTASPVTDDPEQMVEPAVNGAKFVINAAAEAK.
BnaC07g42660D_CCRL  ERLVVYDVDVLDYQSILVSLKTCNAVFCCLDSPEGYDEKEVDLEVRGAINVVEACGRTES

                                      1         10        20        30        40
BnaA07g20090D_CCR2  ..................MRHCHSFVQFIYTDFDFCKHTKKWYCYGNTVAEWETQKVKGV
BnaC06g19540D_CCR2  ......................................................MIGVGS
BnaA02g36250D_CCR2  VKRVVFTSSIGAVYMNPNRDHQTIVDENCWSDLDFCKNTKNWYCYGKMVAEQSAWETAKV
BnaC02g46610D_CCR2  VKRVVFTSSIGAVYMNPNRDPQTIVDESCWSDLDFCKNTKNWYCYGKMVAEQSAWETAKA
BnaA07g35280D_CCR2  VKRVVFTSSIGAVYMNPNRDPQTIVNEDCWSDLDFCKNTKNWYCYGKMVAEQSAWETAKA
BnaC06g40190D_CCR2  VKRVVFTSSIGAVYMNPNRHPQTIVNEDCWSDLDFCKNTKNWYCYGKMVAEQSAWETAKA
BnaA09g56490D_CCR1  VKRVVITSSIGAVYMDPNRDPEAVVDESCWSDLEFCKNTKNWYCYGKMVAEQAAWETAEE
BnaC08g38580D_CCR1  VKRVVITSSIGAVYMDPSRDPEAVVDESCWSDLEFCKNTKNWYCYGKMVAEQAAWETAEE
BnaC05g12180D_CCR1  VKRVVITSSIGAVYMDPNRDPEAVVDESCWSDLEFCKNTKNWYCYGKMVAEQAAWETAKE
BnaA06g10620D_CCR1  VKRVVITSSIGAVYMDPNRDPEAVVDESCWSDLEFCKNTKNWYCYGKMVAEQAAWETAKE
BnaC07g42660D_CCRL  IEKIVFSSSLTASIWRDNIGTQKDVDEKCWSDQDFCRNKKLWHALAKMLSEKAAWALAMD

                             50        60        70        80        90        100
BnaA07g20090D_CCR2  DCRLGGAKSGSSSWNTAPVSDSAVNASLVHILKYLTGSAKTYANLTQVYVNVRNVALGHV
BnaC06g19540D_CCR2  KNLMVMTCEPRFSVVLGTPLQSAVNASLVHILKYLTGSAKTYANLTQVYVDVRNVALGHV
BnaA02g36250D_CCR2  KGVDLVVLNP..VLVLGPPLQSAVNASLVHILKYLTGSAKTYANLTQVYVDVRDVALGHV
BnaC02g46610D_CCR2  KGVDLVVLNP..VLVLGPPLQSAVNASLVHILKYLTGSAKTYANLTQVYVDVRDVALGHV
BnaA07g35280D_CCR2  KGVDLVVLNP..VLVLGPPLQSAVNASLVHILKYLTGSAKTYANLTQVYVDVRDVALGHV
BnaC06g40190D_CCR2  KGVDLVVLNP..VLVLGPPLQSAVNASLVHILKYLTGSAKTYANLTQVYVDVRDVALGHV
BnaA09g56490D_CCR1  KGVDLVVLNP..VLVLGPPLQPTINASLFHVLKYLTGSAKTYANLTQAYVDVRDVALAHV
BnaC08g38580D_CCR1  KGVDLVVLNP..VLVLGPPLQPTINASLFHVLKYLTGSAKTYANLTQAYVDVRDVALAHV
BnaC05g12180D_CCR1  KGVDLVVLNP..VLVLGPPLQPTINASLFHVLKYLTGSAKTYANLTQAYVDVRDVALAHV
BnaA06g10620D_CCR1  KGVDLVVLNP..VLVLGPPLQPTINASLFHVLKYLTGSAKTYANLTQAYVDVRDVALAHV
BnaC07g42660D_CCRL  RRLNMVSINP..GLIVGP...SVAQYNPRPTMSYLKGAAQMYENGVLAYVDVKFLADVHI

                            110
BnaA07g20090D_CCR2  MVYDTQRLRPLHLFQK............................................
BnaC06g19540D_CCR2  MVYQGHN.....................................................
BnaA02g36250D_CCR2  MVYESPSASGRYILAETALHRGEVVEILAKFFPEYPLPTKCSDEKNPRAKPYKFTTQKIK
BnaC02g46610D_CCR2  MVYESPSASGRYILAETALHRGEVVEILAKFFPEYPLPIKCSDEKNPRAKPYKFTTQKIK
BnaA07g35280D_CCR2  MVYESSSASGRYILAETALHRGEVVEILAKFFPEYPLPTKCSDDKNPRAKPYKFTTQKIK
BnaC06g40190D_CCR2  MVYESSSASGRYILAETALHRGEVVEILAKFFPEYPLPTKCSDDKNPRAKPYKFTTQKIK
BnaA09g56490D_CCR1  LVYEAPSASGRYLLAESALHRGEVVEILAKLFPEYPLPTKCKDEKNPRAKPYKFTNQKIK
BnaC08g38580D_CCR1  LVYEAPSASGRYLLAESALHRGEVVEILAKLFPEYPLPTKCKDEKNPRAKPYKFTNQKIK
BnaC05g12180D_CCR1  LVYEAPSASGRYLLAESAFHRGEVVEILAKLFPEYPIPTKCKDEKNPRAKPYKFTNQKIK
BnaA06g10620D_CCR1  LVYEAPTASGRYLLAESALHRGEVVEILAKLFPEYPLPTK....................
BnaC07g42660D_CCRL  RAYEDVSACGRYFCFNQIVNTEEEALKLVESLSPLIPMPPRYESVMQGSEVYEERLRNNK

BnaA07g20090D_CCR2  ...............................................
BnaC06g19540D_CCR2  ...............................................
BnaA02g36250D_CCR2  DLGLEFKPIKQSLYESVKSLQEKGHLPLPQDSNQNVIIES.......
BnaC02g46610D_CCR2  DLGLEFKPIKQSLYESVKSLQEKGHLPLPQDSNQNVIIES.......
BnaA07g35280D_CCR2  DLGLEFTPIKQSLYDSVKSLQEKGHLPLPQYSNQDNVTIES......
BnaC06g40190D_CCR2  DLGLEFTPIKQSLYDSVKSLQEKGHLRLPQYSNQDNVTIES......
BnaA09g56490D_CCR1  DLGLEFTSTKQSLYDTVKSLQEKGHLPPPPPPSTSEGSSQNGIRIES
BnaC08g38580D_CCR1  DLGLEFTSTKQSLYDTVKSLQEKGHLP...PPSTSQESSQNGIKIES
BnaC05g12180D_CCR1  DLGLEFTSTKQSLYDTVKSLQEKGHLPPPPPPSTSQESSQNGIKIES
BnaA06g10620D_CCR1  ...............................................
BnaC07g42660D_CCRL  LSKLVEAGSAC....................................
```

Domain

Fig. S8. Protein sequence alignment analysis between CCRL and CCR in *B. napus*.

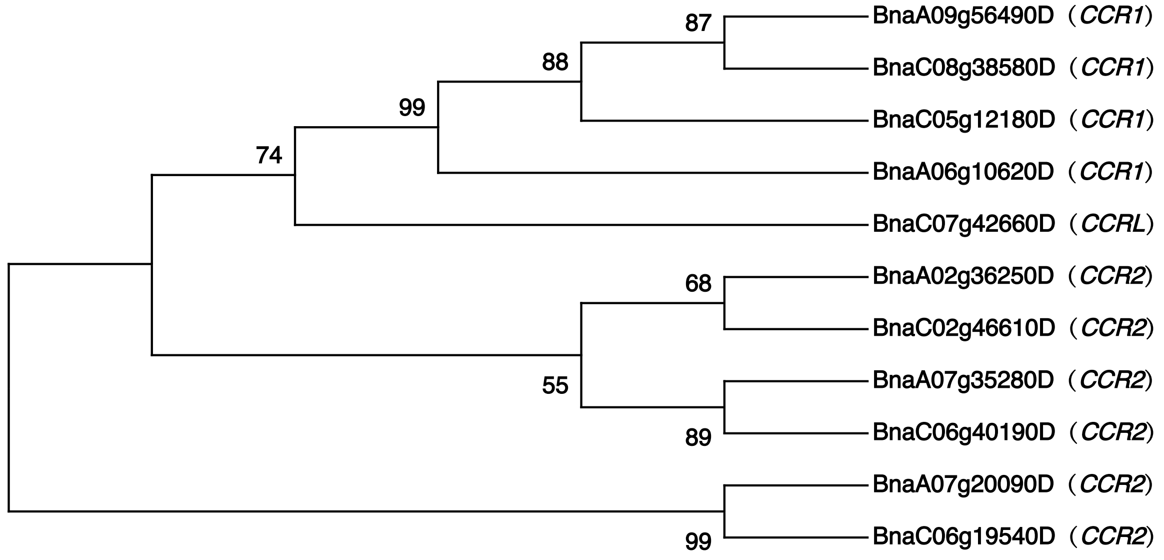

Fig. S9. Phylogenetic tree analysis of *CCRL* and *CCR* genes in *B. napus*.

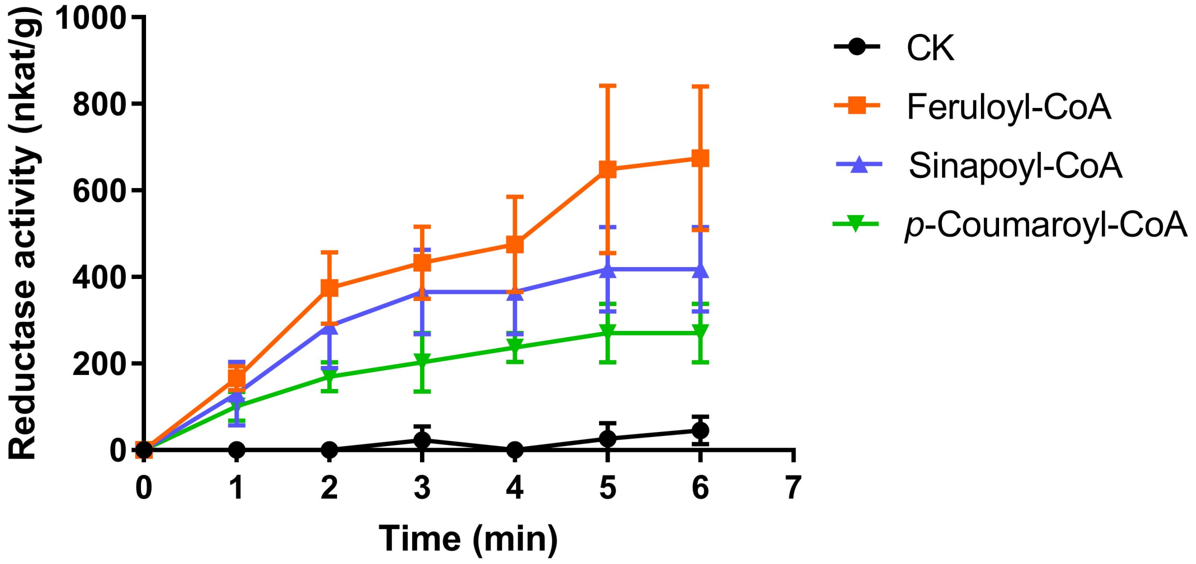

Fig. S10 The reductase activity of recombinant BnaCCRL.

BnaC07.CCRL-sgRNA1

```
L21:GGTGAGAGGAGCTATCAATG--GTGGAAGCATG     (deletion)
L35:GGTGAGAGGAGCTATCAATGTGGTGGAAGCATG     (WT)
L43:GGTGAGAGGAGCTATCAATGGTGGTGGAAGCATG    (insertion)
L48:GGTGAGAGGAGCTATCAATGGTGGTGGAAGCATG    (insertion)
Ref:GGTGAGAGGAGCTATCAATGTGGTGGAAGCATG
```

BnaC07.CCRL-sgRNA2

```
L21:GTCTCCTTTGATACCTATGCCAACCGAGGTACATT   (insertion)
L35:GTCTCCTTTGATACCTATGCCAACCGAGGTACATT   (insertion)
L43:GTCTCCTTTGATACCTATGCCAACCGAGGTACATT   (insertion)
L48:GTCTCCTTTGATACCTATGCCAACCGAGGTACATT   (insertion)
Ref:GTCTCCTTTGATACCTATGCCACCGAGGTACATT
```

BnaA03.CCRL-sgRNA1

```
L21:GGTGAGAGGAGCTATCAATGTTGGTGGAAGCATG  (insertion)
L35:GGTGAGAGGAGCTATCAATGTTGGTGGAAGCATG  (insertion)
L43:GGTGAGAGGAGCTATCAATGTTGGTGGAAGCATG  (insertion)
L48:GGTGAGAGGAGCTATCAATGTTGGTGGAAGCATG  (insertion)
Ref:GGTGAGAGGAGCTATCAATGTGGTGGAAGCATG
```

BnaA03.CCRL-sgRNA2

```
L21:GTCTCCTTTGATACCTATGCCAACCGAGGTACATT (insertion)
L35:GTCTCCTTTGATACCTATGCCCACCGAGGTACATT (insertion)
L43:GTCTCCTTTGATACCTATGCCAACCGAGGTACATT (insertion)
L48:GTCTCCTTTGATACCTATGTCCACCGAGGTACATT (insertion)
Ref:GTCTCCTTTGATACCTATGCCACCGAGGTACATT
```

BnaC05.CCRL-sgRNA1

```
L21:GGTGAGAGGAGCTATCAATGTTGGTGGAAGCATG  (insertion)
L35:GGTGAGAGGAGCTATCAATGTGGTGGAAGCATG   (WT)
L43:GGTGAGAGGAGCTATCAATGTTGGTGGAAGCATG  (insertion)
L48:GGTGAGAGGAGCTATCAATGTGGTGGAAGCATG   (WT)
Ref:GGTGAGAGGAGCTATCAATGTGGTGGAAGCATG
```

Fig. S11 Genotype of *BnaCCRL-sgRNA* mutant lines in the T_0 generation. Nucleotides are shown with sgRNA target (red), PAM sequence (green) and mutation sites (blue) indicated.

BnaC07.CCRL

```
L21:LEVRGAINGGSMWKNREHREDCILFFFNSFNLERQHWHSEGCEVLESRLLS 150
L35:LEVRGAINVVEACGRTESIEKIVFSSSLTASIWRDNIGTQKDVDEKCWSDQ 150
L43:LEVRGAINVVEACGRTESIEKIVFSSSLTASIWRDNIGTQKDVDEKCWSDQ 150
L48:LEVRGAINVVEACGRTESIEKIVFSSSLTASIWRDNIGTQKDVDEKCWSDQ 150
Ref:LEVRGAINVVEACGRTESIEKIVFSSSLTASIWRDNIGTQKDVDEKCWSDQ 150

L21:QQKVVARIGKDVVRESSLGTSHGPAQHGLYQSWSYRWTIGCTIPAHHVLP  200
L35:DFCRNKKLWHALAKMLSEKAAWALAMDRRLNMVSINPGLIVGPSVAQYNP  200
L43:DFCRNKKLWHALAKMLSEKAAWALAMDRRLNMVSINPGLIVGPSVAQYNP  200
L48:DFCRNKKLWHALAKMLSEKAAWALAMDRRLNMVSINPGLIVGPSVAQYNP  200
Ref:DFCRNKKLWHALAKMLSEKAAWALAMDRRLNMVSINPGLIVGPSVAQYNP  200

L21:RSCTNVEWSVGVRRRIFSGCSYSIRGCFSLWSIFLLQPNRHRRRSSQACR  250
L35:RPTMSYLKGAAQMYENGVLAYVDVKFLADVHIRAYEDVSACGRYFCFNQI  250
L43:RPTMSYLKGAAQMYENGVLAYVDVKFLADVHIRAYEDVSACGRYFCFNQI  250
L48:RPTMSYLKGAAQMYENGVLAYVDVKFLADVHIRAYEDVSACGRYFCFNQI  250
Ref:RPTMSYLKGAAQMYENGVLAYVDVKFLADVHIRAYEDVSACGRYFCFNQI  250

L21:EFVSFDTYANRGMRVCKEAKFTKKDGTINASWWKLALLV* 289
L35:VNTEEEALKLVESLSPLIPMPTEVECNARKRSLRRKTEEQIEQAGGSWLC  300
L43:VNTEEEALKLVESLSPLIPMPTEVECNARKRSLRRKTEEQIEQAGGSWLC  300
L48:VNTEEEALKLVESLSPLIPMPTEVECNARKRSLRRKTEEQIEQAGGSWLC  300
Ref:VNTEEEALKLVESLSPLIPMPPRYESVMQGSEVYEERLRNNKLSKLVEAG  300

L35:LL*  302
L43:LL*  302
L48:LL*  302
Ref:SAC* 303
```

BnaA03.CCRL

```
L21:LEVRGAINVGGSMWKNREHREDCILFFFNSFNLERQHWNSEGCEVLESRLL 150
L35:LEVRGAINVGGSMWKNREHREDCILFFFNSFNLERQHWNSEGCEVLESRLL 150
L43:LEVRGAINVGGSMWKNREHREDCILFFFNSFNLERQHWNSEGCEVLESRLL 150
L48:LEVRGAINVGGSMWKNREHREDCILFFFNSFNLERQHWNSEGCEVLESRLL 150
Ref:LEVRGAINVVEACGRTESIEKIVFSSSLTASIWRDNIGTQKDVDEKCWSDQ 150

L21:SQQKVVARIGKDVVRESSLGTSHGPAQHGLYQSWSYRWTIGCTIPAHHVL  200
L35:SQQKVVARIGKDVVRESSLGTSHGPAQHGLYQSWSYRWTIGCTIPAHHVL  200
L43:SQQKVVARIGKDVVRESSLGTSHGPAQHGLYQSWSYRWTIGCTIPAHHVL  200
L48:SQQKVVARIGKDVVRESSLGTSHGPAQHGLYQSWSYRWTIGCTIPAHHVL  200
Ref:DFCRNKKLWHALAKMLSEKAAWALAMDRRLNMVSINPGLIVGPSVAQYNP  200

L21:PQRSCTNVEWSVGVRRRIFSGCSYSIRGCFSLWSIFLLQPNRHRRRSSQA  250
L35:PQRSCTNVEWSVGVRRRIFSGCSYSIRGCFSLWSIFLLQPNRHRRRSSQA  250
L43:PQRSCTNVEWSVGVRRRIFSGCSYSIRGCFSLWSIFLLQPNRHRRRSSQA  250
L48:PQRSCTNVEWSVGVRRRIFSGCSYSIRGCFSLWSIFLLQPNRHRRRSSQA  250
Ref:RPTMSYLKGAAQMYENGVLAYVDVKFLADVHIRAYEDVSACGRYFCFNQI  250

L21:CREFVSFDTYATEVECNARKRSLRRKTEEQIEQAGGSWLCLL* 292
L35:CREFVSFDTYATEVECNARKRSLRRKTEEQIEQAGGSWLCLL* 292
L43:CREFVSFDTYATEVECNARKRSLRRKTEEQIEQAGGSWLCLL* 292
L48:CREFVSFDTYATEVECNARKRSLRRKTEEQIEQAGGSWLCLL* 292
Ref:VNTEEEALKLVESLSPLIPMPPRYESVMQGSEVYEERLRNNKLSKLVEAG  300

Ref:SAC* 303
```

BnaC05.CCRL

```
L21:RGAINVGGSMKNREHRDCVLFFFFNSFNFERQHWNSEGCEVLESRLLSQQ 100
L35:RGAINVVEACERTESIDKIVFSSSSLTASILRDNIGTQKDVDEKCWSDQD 100
L43:RGAINVVEACERTESIDKIVFSSSSLTASILRDNIGTQKDVDEKCWSDQD 100
L48:RGAINVVEACERTESIDKIVFSSSSLTASILRDNIGTQKDVDEKCWSDQD 100
Ref:RGAINVVEACERTESIDKIVFSSSSLTASILRDNIGTQKDVDEKCWSDQD 100

L21:KVCRRNGRVYWIAGGCERYLSYQRHDVKCLCCLESREASNYCSTSKNCCE 150
L35:FCRNKKFAEGMVEFIGLLEVAVKKGTNLAIRDMMSSDAYVALNLGKQVTI 150
L43:FCRNKKFAEGMVEFIGLLEVAVKKGTNLAIRDMMSSDAYVALNLGKQVTI 150
L48:FCRNKKFAEGMVEFIGLLEVAVKKGTNLAIRDMMSSDAYVALNLGKQVTI 150
Ref:FCRNKKFAEGMVEFIGLLEVAVKKGTNLAIRDMMSSDAYVALNLGKQVTI 150

L21:LFEPGMESRTHAVCSQLWSCKTASVLYILCWHNGRSSTYPASDNICDGVW 200
L35:VHKLQKTVVNCNLNPVWNQELMLSVPDSYGPVKLQVYDYDTFSAGDIMGE 200
L43:VHKLQKTVVNCNLNPVWNQELMLSVPDSYGPVKLQVYDYDTFSAGDIMGE 200
L48:VHKLQKTVVNCNLNPVWNQELMLSVPDSYGPVKLQVYDYDTFSAGDIMGE 200
Ref:VHKLQKTVVNCNLNPVWNQELMLSVPDSYGPVKLQVYDYDTFSAGDIMGE 200

L21:GSRDVWGYADWEMAKVASANRQHHL* 225
L35:AQLDIQPLITSAMVFGDPEMFGDMQIGKWLKLHDNPPIDDSII* 243
L43:AQLDIQPLITSAMVFGDPEMFGDMQIGKWLKLHDNPPIDDSII* 243
L48:AQLDIQPLITSAMVFGDPEMFGDMQIGKWLKLHDNPPIDDSII* 243
Ref:AQLDIQPLITSAMVFGDPEMFGDMQIGKWLKLHDNPPIDDSII* 243
```

Fig. S12 Amino acid sequence alignment of the target sites derived from WT and *BnaCCRL-sgRNA* lines.

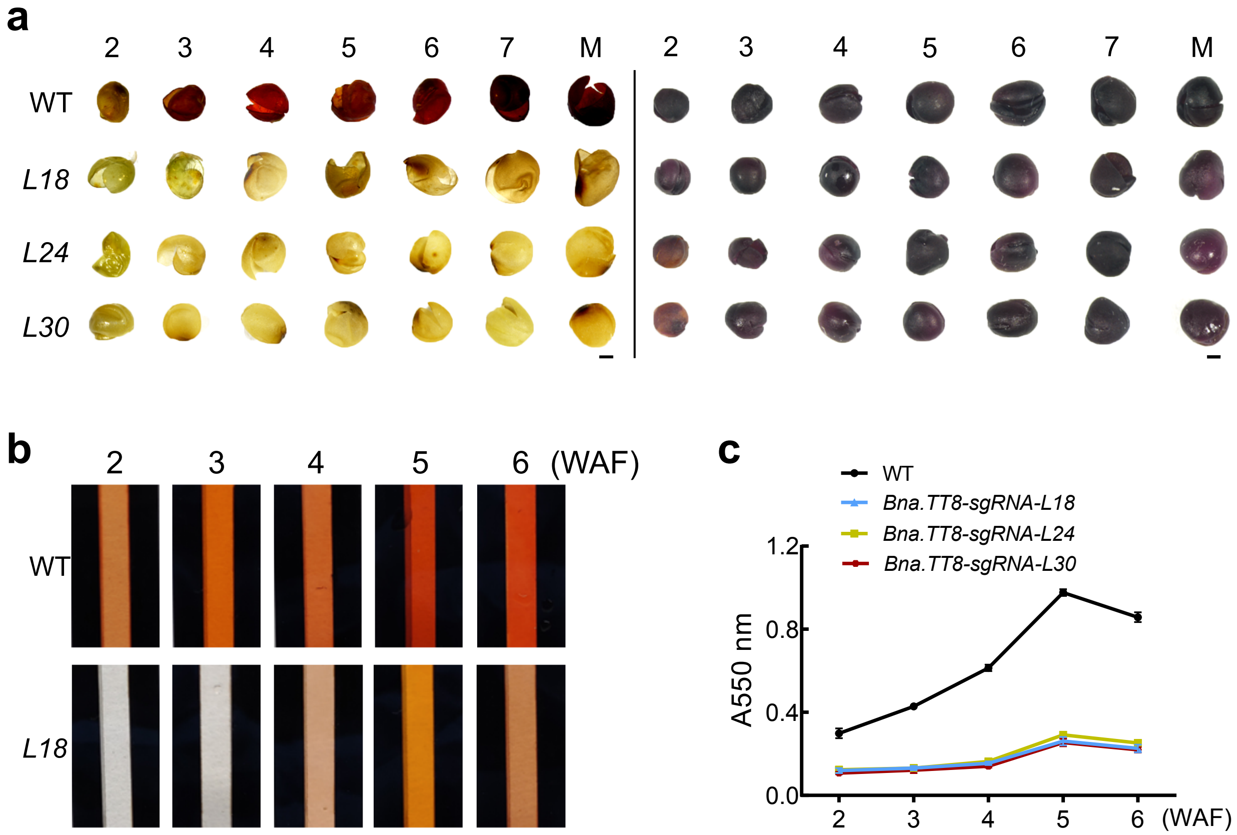

Fig. S13 Chemical staining of procyanidin in seed coats and embryos of the *BnaTT8* mutants (L18, L24 and L30) and the control at 2 weeks after flowering (WAF), 3 WAF, 4 WAF, 5 WAF, 6 WAF, 7 WAF and mature seed and estimation of insoluble procyanidin oligomers (PCs).

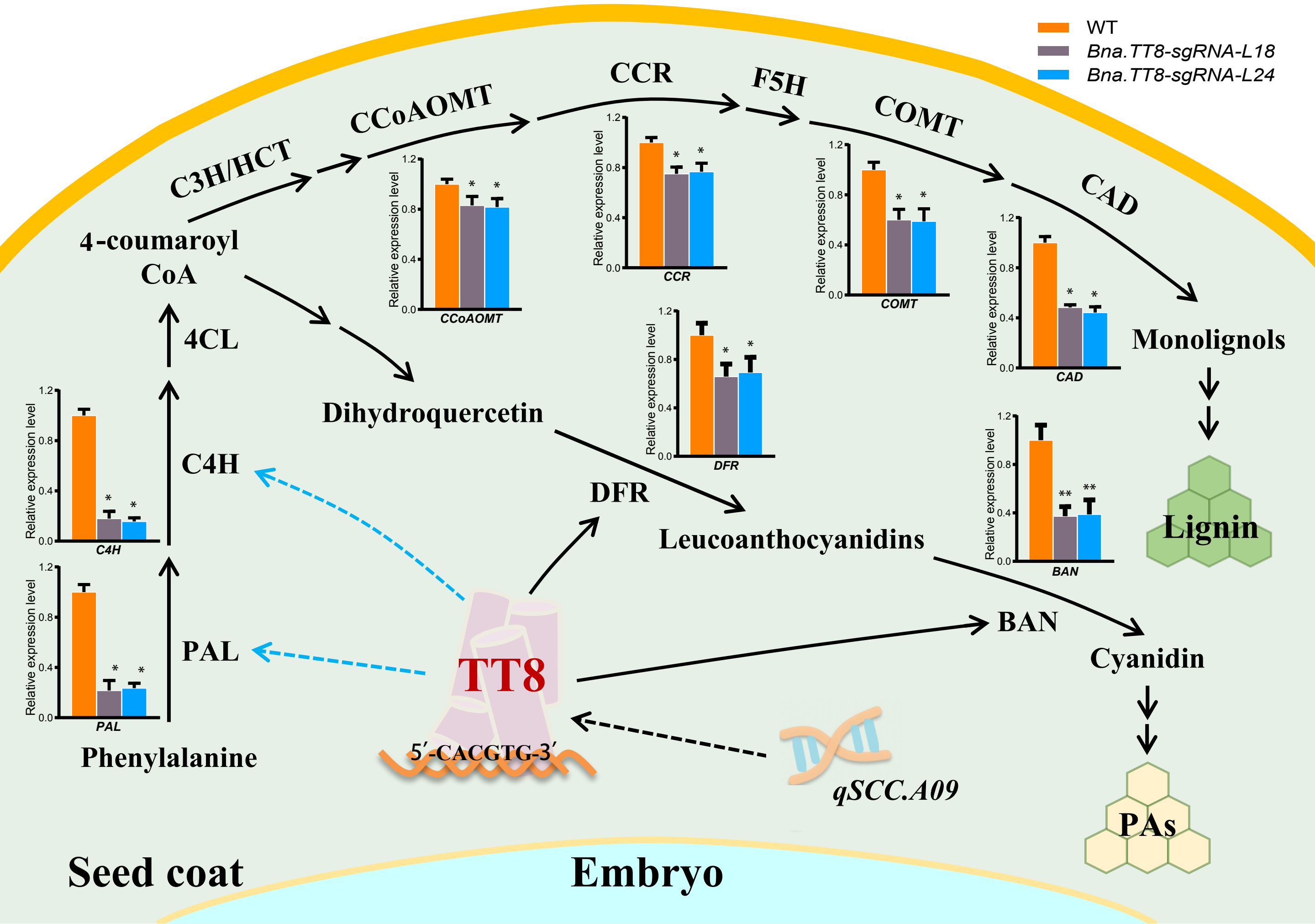

Fig. S14 A proposed working model of *BnaTT8* in the regulation of SCC. Expression levels of procyanidin and lignin biosynthesis genes determined by qRT-PCR in the phenylpropanoid pathway in the *BnaTT8* double knockout mutant seeds. Values are means ± SE of three biological replicates (n = 3). Student's t-test was used for statistical analysis between the *BnaTT8-sgRNA* lines and WT (*, P < 0.05; **, P < 0.01). Blue dashed lines represent indirect regulation, black line represents direct regulation or known metabolic pathways. Black dashed line represents putative regulation. TT8, Transparent Testa 8; PAL, phenylalanine ammonia-lyase; C4H, cinnamate 4-hydroxylase; 4CL, 4-coumarate-CoA ligase; C3H, coumarate 3-hydroxylase; HCT, hydroxycinnamoyl-CoA shikimate/quinate hydroxycinnamoyl transferase; CCoAOMT, caffeoyl-CoA O-methyltransferase; CCR, cinnamoyl-CoA reductase; F5H, ferulate 5-hydroxylase; COMT, caffeic acid O-methyltransferase; CAD, cinnamyl alcohol dehydrogenase; DFR, dihydroflavonol-4-reductase; BAN, BANYULS.

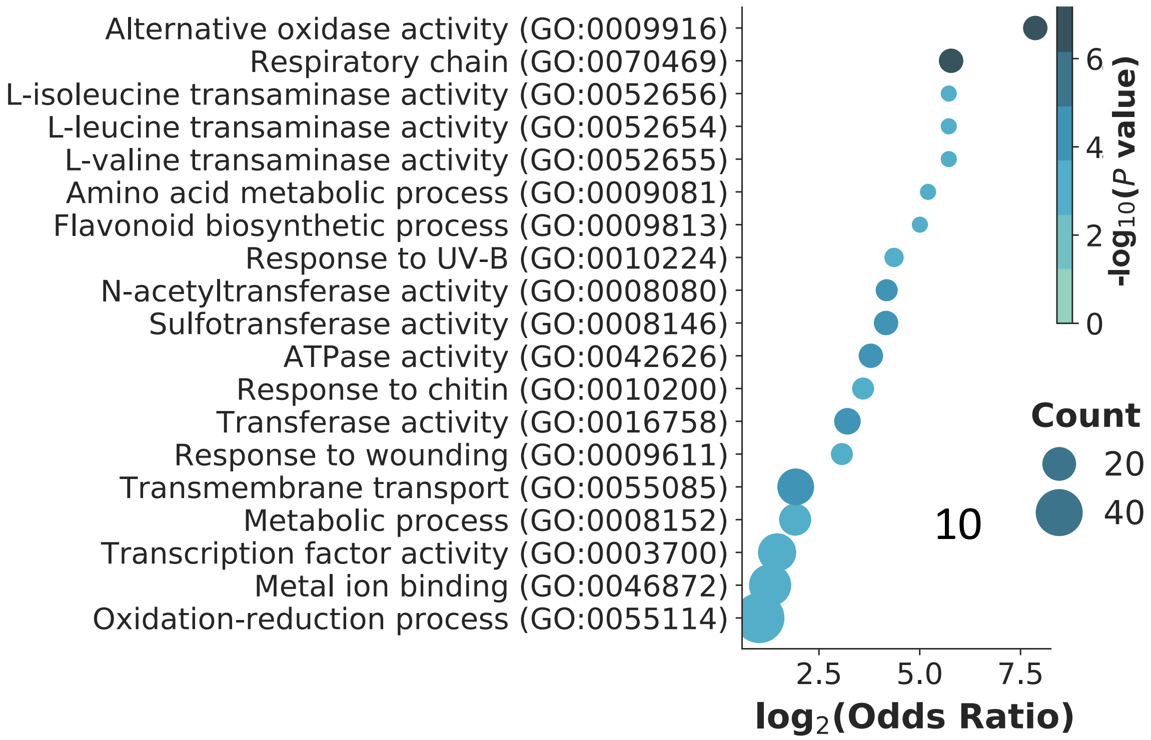

Fig. S15 GO enrichment analysis of overlapped significant genes identified by TWAS for SOC and SCC. The dot size and color indicate the gene number and the range of FDR values, respectively.

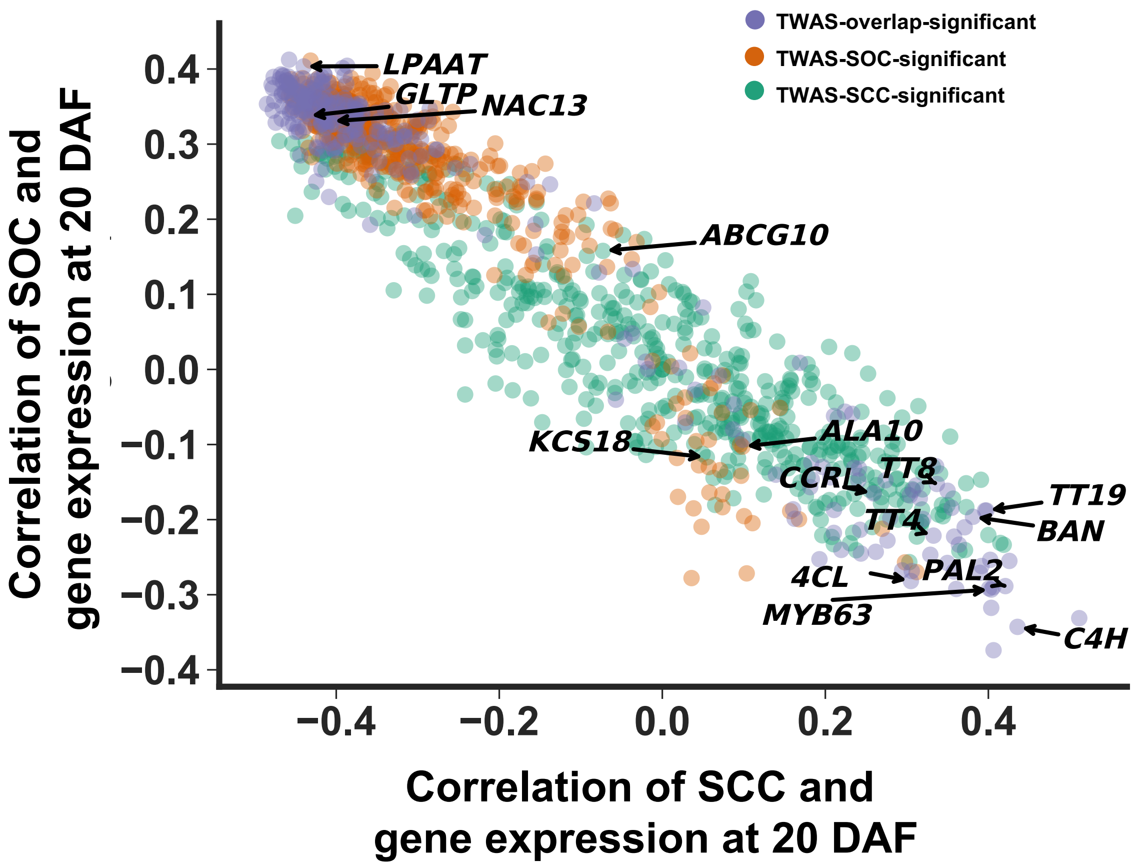

Fig. S16 Correlation analysis between TWAS significant gene expression and SOC trait or SCC trait at 20 DAF. Each dot represents a gene.

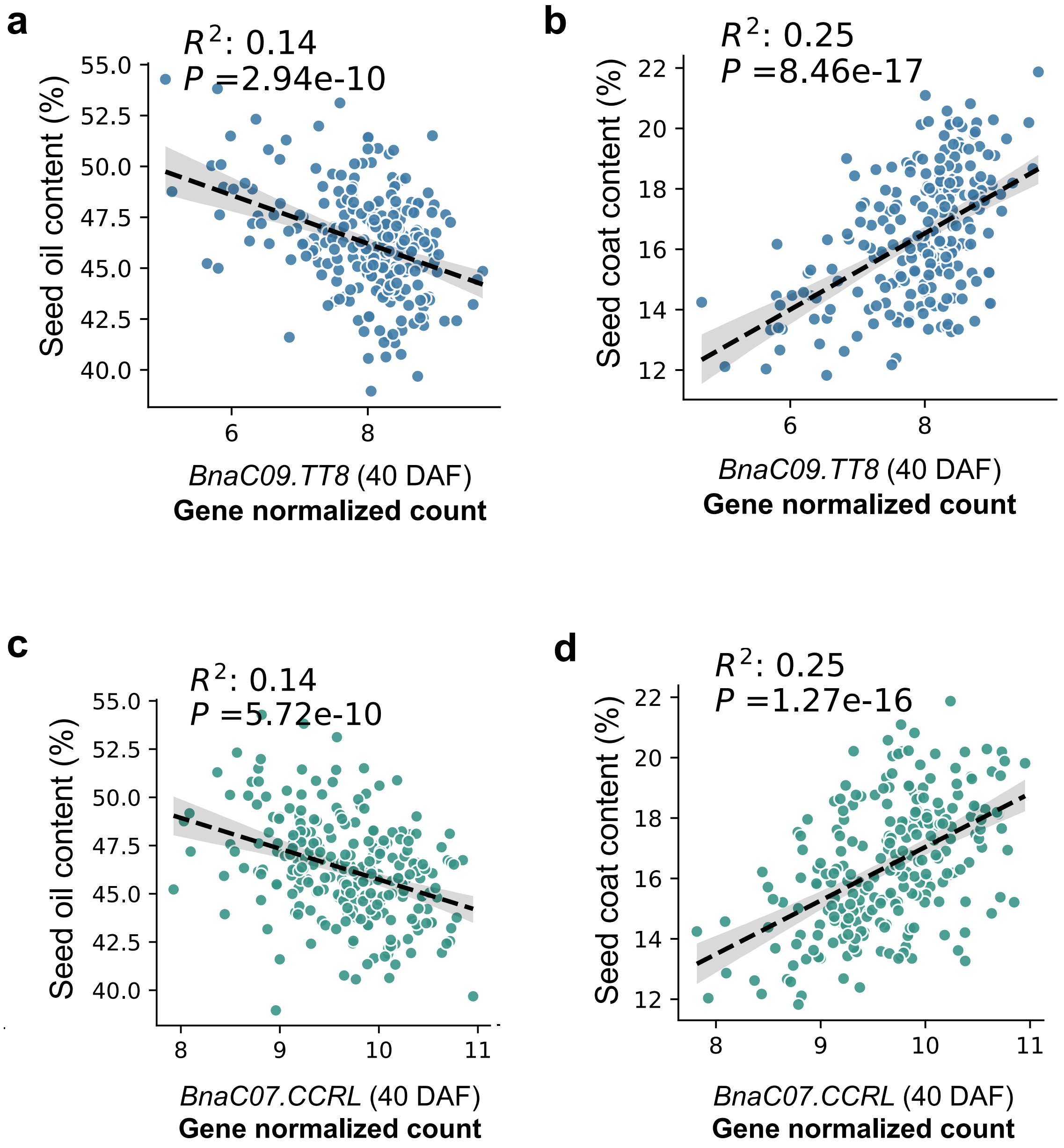

Fig. S17 Correlation between SOC and expression levels of *BnaC09.TT8* at 40 DAF (a), SCC and expression levels of *BnaC09.TT8* at 40 DAF (b), SOC and expression levels of *BnaC07.CCRL* at 40 DAF (c) and SCC and expression levels of *BnaC07.CCRL* at 40 DAF (d). Each point in a-d represents an accession. The normalized gene expression values are plotted on the X-axis and the SOC or SCC corresponding to the accessions is plotted on the Y-axis.

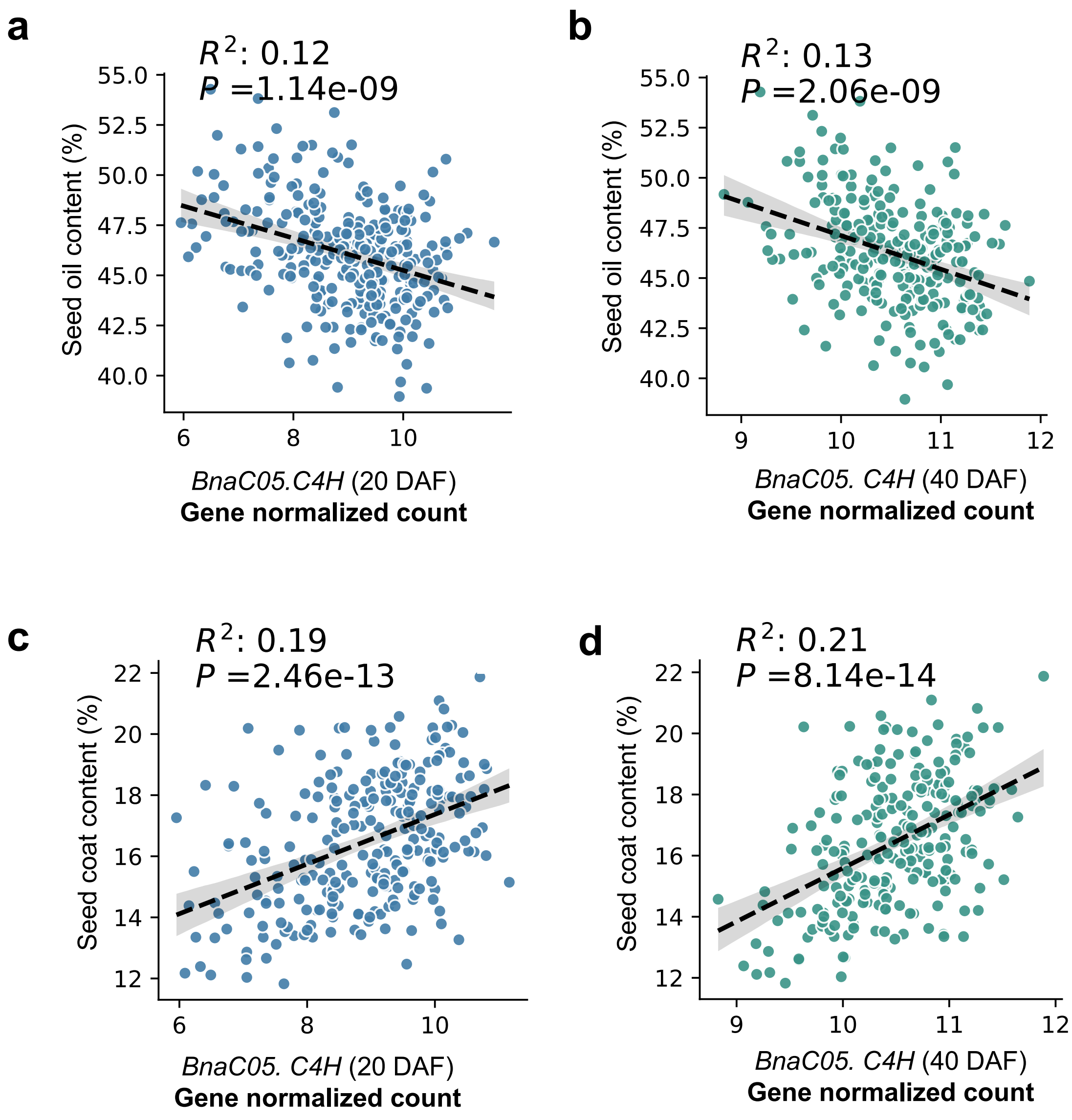

Fig. S18 Correlation between SOC and expression levels of *BnaC05.C4H* at 20 DAF (a), SOC and expression levels of *BnaC05.C4H* at 40 DAF (b), SCC and expression levels of *BnaC05.C4H* at 20 DAF (c) and SCC and expression levels of *BnaC05.C4H* at 40 DAF (d). Each point in a-d represents an accession. The normalized gene expression values are plotted on the X-axis and the SOC or SCC corresponding to the accessions is plotted on the Y-axis.

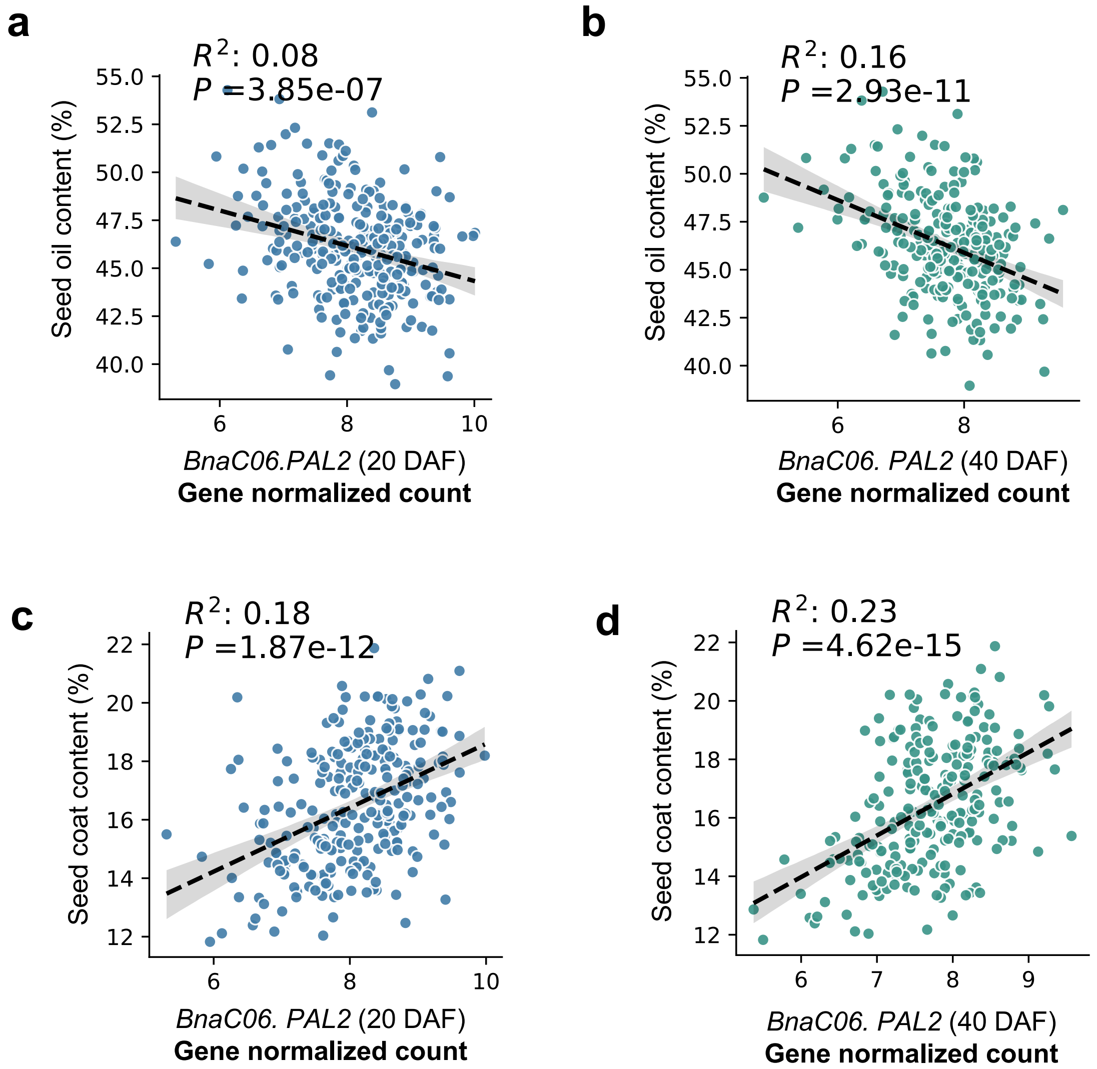

Fig. S19 Correlation between SOC and expression levels of *BnaC06.PAL2* at 20 DAF (a), SOC and expression levels of *BnaC06.PAL2* at 40 DAF (b), SCC and expression levels of *BnaC06.PAL2* at 20 DAF (c) and SCC and expression levels of *BnaC06.PAL2* at 40 DAF (d).

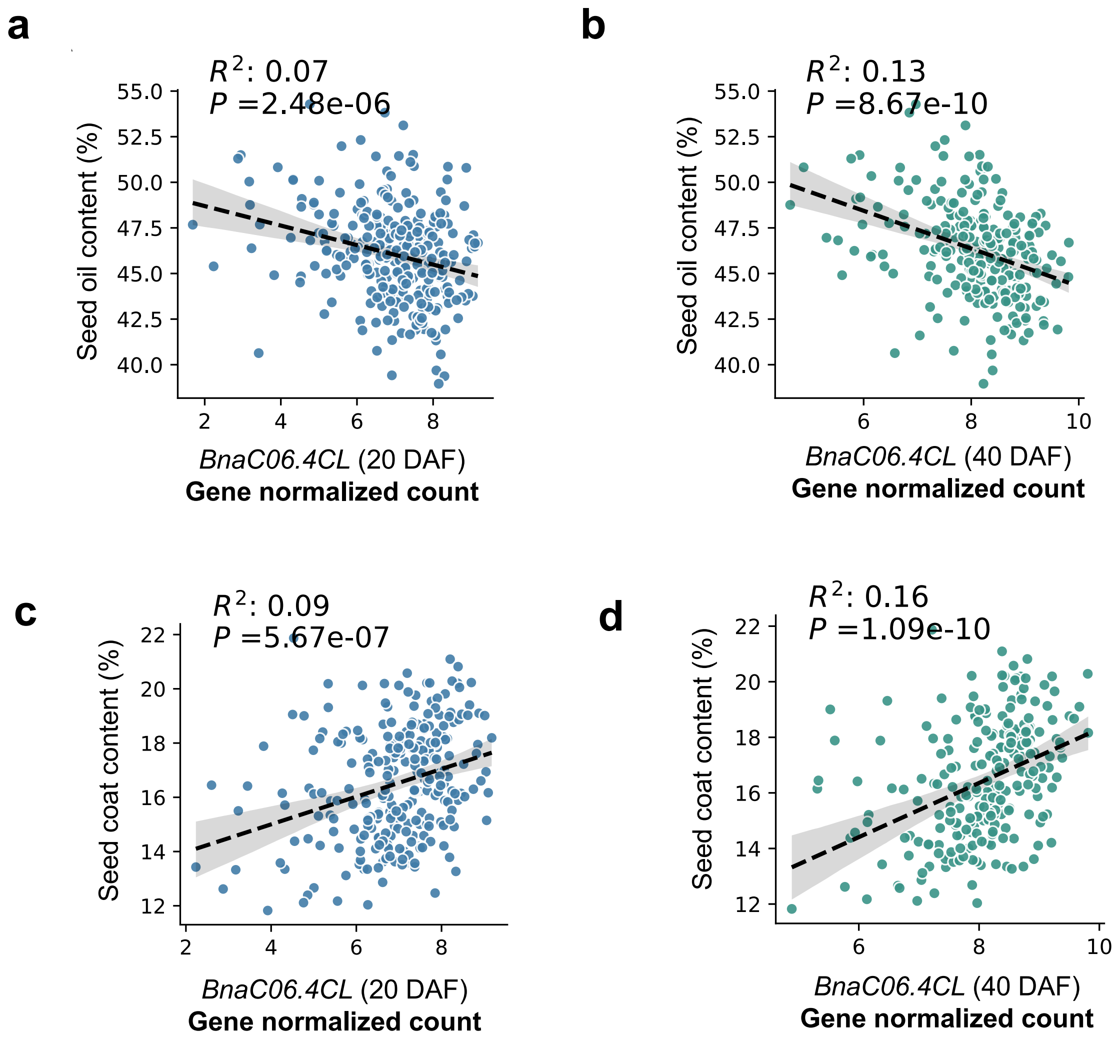

Fig. S20 Correlation between SOC and expression levels of *BnaC06.4CL* at 20 DAF (a), SOC and expression levels of *BnaC06.4CL* at 40 DAF (b), SCC and expression levels of *BnaC06.4CL* at 20 DAF (c) and SCC and expression levels of *BnaC06.4CL* at 40 DAF (d).
