# Supplementary material for: Multi-omics analysis dissects the genetic architecture of seed coat content in Brassica napus
Source: Genome Biol. 2022 Mar 28;23:86. doi: 10.1186/s13059-022-02647-5 (PMC8962237; doi:10.1186/s13059-022-02647-5)
Supplement: Supplementary file 3 — Additional file 3. Review history. [file 13059_2022_2647_MOESM3_ESM.docx]

Review History

**First round of review**

**Reviewer 1**

**Are you able to assess all statistics in the manuscript, including the appropriateness of statistical tests used?**

Yes, and I have assessed the statistics in my report.

**Comments to author:**

Brassica napus is an important oil crop. By transcriptome-wide association studies (TWAS) and genome-wide association studies (GWAS) using 382 B. napus accessions, the authors identified the genes associated with seed coat content (SOC), which negatively correlates with seed oil content. The authors claimed that BnaC07.CCRL and BnaTT8s played key roles in the determination of SOC, presumably by regulating lignin biosynthesis.

TT8 is well characterized as one of the components of the MBW complex regulating flavonoid biosynthesis. It is interesting that TT8 seems to regulate the expression of many lignin biosynthetic genes. However, more evidence is required to make such conclusion. For instance, does TT8 share a similar expression pattern with the lignin biosynthetic genes? Are those genes direct targets? The authors could try a transient assay to see if TT8 can activate the promoters of those potential target genes.

CCRL may not the real Cinnamoyl-CoA reductase. The authors should prove the enzyme activity.

**Reviewer 2**

**Are you able to assess all statistics in the manuscript, including the appropriateness of statistical tests used?**

Yes, and I have assessed the statistics in my report.

**Comments to author:**

The manuscript entitled "A multi-omics analysis dissects the genetic architecture of seed coat content in Brassica napus" (Manuscript number: GBIO-D-21-01846) has skillfully employed a multi-omics analysis method including GWAS, TWAS, QTL mapping within large natural population to uncover the key modules and key genes that highly related (negative or positive) to SCC (Seed Coat Content) of B. napus. Then, the authors have selected the key genes BnaCCR-like and BnaTT8 for functions validation by CRISPR/Cas9 system and have discovered that both of these genes positively regulate SCC and lignin content. Furthermore, the authors have also evaluated the key genes regulating carbon allocation between oil and phenylpropane compounds during the seeds development of B. napus. Taken together, this study will fascinate us in understanding the underlying machinery of seed coat development and provide a valuable genetic resource for genetic improvement of SCC to breed B. napus seeds with high oil content. Here are some questions or some places that the authors should answer or care about.

1. Why the authors selected 20 (DAF) days after flowering and 40 DAF as the developmental stage for experiments rather than other stages in this study? Please clearly clarify the reasons in the main text.

2. Previous paper "Targeted mutagenesis of BnTT8 homologs controls yellow seed coat development for effective oil production in Brassica napus L."published in PBJ has already applied CRISPR/Cas9 for creating yellow-seeded mutants in rapeseed. And this work also has discovered that BnTT8 double mutants generated yellow seeds with elevated seed oil and the targeted mutations of BnTT8 resulted in the broad suppression of phenylpropanoid/flavonoid biosynthesis genes. Whether the editing sites of yours are different with that of in this paper? If different, please describe in main text.

3. A previous paper entilted"Targeted knockout of BnTT2 homologues for yellow-seeded Brassica napus with reduced flavonoids and improved fatty acid composition"has found that the BnTT2 mutation can be used for yellow seed breeding and oil improvement. And in B. napus, TT family genes contain TT2, TT4, TT5, TT8 and TT12. Therefore, there is a question why solely knockout TT2 or TT8 can both confer yellow seed phenotype and with high oil content in B. napus? How about the relationship between TT2 and TT8?

4. For the TWAS-SCC significant genes identified in Fig1, all the genes labeled are positive genes, how about the negative genes? What are the functions of the negative genes?

5. Is the gene CCR-LIKE an unreported gene? Can you add some gene evolutionary tree analysis and analysis of conserved structural domains of the gene? You should compare the CCR-LIKE and CCR in B. napus.

6. The regulatory network of key genes for carbon source allocation is shown in Fig6c. How many of the SOC-TWAS significant associated genes are regulated by the A09 hotspot?

7. TWAS significant genes in eGWAS localized to the A09 region are mainly enriched in many categories related to phenylpropanoid metabolism and abiotic stresses". What are the features of other genes that are not localized to the A09 region? Are they enriched in some other pathways?

8. Is it possible to predict some key genes or transcription factors that regulate genes related to seed coat synthesis in the A09 hotspot region?

9. The oil in rapeseeds is mostly concentrated in the embryo, is there any data on the ratio of embryo to seed weight in the population, can you do some analysis that may be more related data on carbon source allocation between SCC and ratio of embryo to seed weight rather than SOC?

Minor points:

1. The names of TT8 and CCRL should be highlighted in Fig2e.

2. Fig3a should be clearly labeled with 3 significant QTLs and their names.

3. The SNP markers in Fig3c should be marked with more distinctive colors and the eQTLs should be clearly labeled as to which gene corresponds to the eQTL.

4. Whether a histogram can be drawn for the hotspot regulatory regions identified in the whole genome, instead of just a dot plot.

5. Since the modules identified in the study that were significantly negatively correlated with SCC, the correlation analysis between them and SOC should be added.

**Authors Response**

**Point-by-point responses to the reviewers’ comments:**

Reviewer #1:

Brassica napus is an important oil crop. By transcriptome-wide association studies (TWAS) and genome-wide association studies (GWAS) using 382 B. napus accessions, the authors identified the genes associated with seed coat content (SOC), which negatively correlates with seed oil content. The authors claimed that BnaC07.CCRL and BnaTT8s played key roles in the determination of SOC, presumably by regulating lignin biosynthesis.

1. TT8 is well characterized as one of the components of the MBW complex regulating flavonoid biosynthesis. It is interesting that TT8 seems to regulate the expression of many lignin biosynthetic genes. However, more evidence is required to make such conclusion. For instance, does TT8 share a similar expression pattern with the lignin biosynthetic genes? Are those genes direct targets? The authors could try a transient assay to see if TT8 can activate the promoters of those potential target genes.

*Response 1: Thank you for your suggestion. We proposed and discussed that TT8 indirectly impacted the expression of many lignin biosynthetic genes based on multiple bioinformatics and experimental evidences (Fig. 3c-3d, Fig. 4e, Fig. S4, Fig. S5 and Fig. S14). We have performed further analysis to address your questions.*

*(1) BnaTT8s showed a similar expression pattern with some lignin synthesis-related genes. The analysis revealed that BnaTT8s expression was correlated with the expression of CAD4, CAD5, CCR1, CCR2, CCoAOMT1 and several other lignin-related genes, suggesting BnaTT8s affects the synthesis of lignin.*

*In addition, in our main text, we consider that the qSCC.A09 may regulate the expression of these six genes (BnaA03.C4H, BnaA05.PAL1, BnaA05.C4H, BnaC03.C4H, BnaC06.PAL2 and BnaC07.CCRL) involved in the general phenylpropanoid pathway and lignin biosynthesis through BnaTT8s (Fig 3d). We performed an expression correlation analysis of BnaTT8s with these six genes and found that BnaTT8s showed a significant correlation with these genes at both 20 DAF and 40 DAF (Fig. S4 and S5).*

*(2) The lignin biosynthetic genes are unlikely to be the direct targets of BnaTT8s.*

*We performed the quantitative PCR to analyze the expression levels of the lignin biosynthetic genes in developing seeds of BnaTT8 mutants and found that the expression of PAL, C4H, CCoA*OMT, CCR, *COMT and CAD were downregulated when BnaTT8s were knockout, suggesting that BnaTT8s may play a positive role in lignin biosynthesis (Fig. S14). TT8 is a transcription factor of bHLH family. To further analyze whether lignin biosynthetic genes are the direct downstream target genes of BnaTT8s, we performed motif enrichment analysis using Fimo (https://meme-suite.org/meme/tools/fimo), and no TT8 binding motif was found in the promoter region of these lignin synthesis genes. We also analyzed lignin genes’ eQTL and none of the eQTLs contain BnaTT8s. Therefore, the lignin biosynthetic genes are unlikely to be the direct targets of BnaTT8s and we also didn’t try the transient assay. BnaTT8s is likely to indirectly impact the expression of lignin synthesis genes. This part has been described and discussed in line 380-385.*

2. CCRL may not the real Cinnamoyl-CoA reductase. The authors should prove the enzyme activity.

*Response 2: Thanks for your suggestion. We have carried out experiments to determine whether CCRL has enzyme activity. The results showed that CCRL had a reductase activity like CCR when p-coumaroyl-CoA, sinapoyl-CoA or feruloyl-CoA was used as a substrate (Fig. S10). This is also the first report on CCRL’s biochemical activity in plants, supporting its role in lignin synthesis.*

Reviewer #2:

The manuscript entitled "A multi-omics analysis dissects the genetic architecture of seed coat content in Brassica napus" (Manuscript number: GBIO-D-21-01846) has skillfully employed a multi-omics analysis method including GWAS, TWAS, QTL mapping within large natural population to uncover the key modules and key genes that highly related (negative or positive) to SCC (Seed Coat Content) of B. napus. Then, the authors have selected the key genes BnaCCR-LIKE (CCRL) and BnaTT8 for functions validation by CRISPR/Cas9 system and have discovered that both of these genes positively regulate SCC and lignin content. Furthermore, the authors have also evaluated the key genes regulating carbon allocation between oil and phenylpropane compounds during the seeds development of B. napus. Taken together, this study will fascinate us in understanding the underlying machinery of seed coat development and provide a valuable genetic resource for genetic improvement of SCC to breed B. napus seeds with high oil content. Here are some questions or some places that the authors should answer or care about.

1. Why the authors selected 20 (DAF) days after flowering and 40 DAF as the developmental stage for experiments rather than other stages in this study? Please clearly clarify the reasons in the main text.

*Response 1: Thank you for your comments. 20 DAF and 40 DAF are two representative periods of early and late oil biosynthesis in rapeseeds. Seed oil biosynthesis starts approximately at 20 DAF and accumulates actively at 40 DAF in B. napus. We have previously published our study on genetic basis of seed oil content at these two developmental stages [3]. We have added description of the reason for choosing 20 DAF and 40 as the developmental stages in the main text in line 109-112.*

2. Previous paper "Targeted mutagenesis of BnTT8 homologs controls yellow seed coat development for effective oil production in Brassica napus L." published in PBJ has already applied CRISPR/Cas9 for creating yellow-seeded mutants in rapeseed. And this work also has discovered that BnTT8 double mutants generated yellow seeds with elevated seed oil and the targeted mutations of BnTT8 resulted in the broad suppression of phenylpropanoid/flavonoid biosynthesis genes. Whether the editing sites of yours are different with that of in this paper? If different, please describe in main text.

*Response 2: Thank you for your suggestion. The editing sites of BnTT8 genes in this study are indeed different from that in the previous study published in PBJ. In the previous study, the editing sites of BnTT8 genes were located in the 1st, 2nd exons in MIR domain and 4th exon in WD/AD domain, whereas the editing sites of BnTT8 genes in this study were located in the 7th exon in bHLH domain. Due to the different editing sites, we obtained the BnTT8 mutants with different seed coat colors, including variegated seeded BnaTT8-sgRNA lines (L14, L17 and L21) and yellow seeded BnaTT8-sgRNA lines (L18, L24 and L30). Different from the previous study, our results suggest that BnTT8s are master regulators controlling seed coat content by affecting lignin biosynthesis through the general phenylpropane pathway and revealed the carbon source allocation between phenylpropanoid pathway and oil synthesis pathway. The difference of these two studies has been discussed in line 404-408.*

3. A previous paper entitled "Targeted knockout of BnTT2 homologues for yellow-seeded Brassica napus with reduced flavonoids and improved fatty acid composition" has found that the BnTT2 mutation can be used for yellow seed breeding and oil improvement. And in B. napus, TT family genes contain TT2, TT4, TT5, TT8 and TT12. Therefore, there is a question why solely knockout TT2 or TT8 can both confer yellow seed phenotype and with high oil content in B. napus? How about the relationship between TT2 and TT8?

*Response 3: The genes that are mutated to cause testa transparency are collectively referred to as TT (Transparent testa) family genes in Arabidopsis. The stable ternary complex formed by TT2, TT8 and TTG1 is one of the most important regulatory elements in flavonoid biosynthesis, which can directly activate the expression of BAN (BANYULS) and DFR (Dihydroflavonol-4-reductase) and control anthocyanin synthesis [1, 2]. Therefore, TT2 or TT8 mutation can disrupt the function of the ternary complex and thus the accumulation of proanthocyanidins is affected, which will lead to yellow seeds.*

4. For the TWAS-SCC significant genes identified in Fig 1, all the genes labeled are positive genes, how about the negative genes? What are the functions of the negative genes?

*Response 4: Thanks for your suggestion. We combined the results of TWAS-SCC at 20 DAF and 40 DAF and identified a total of 161 negative association genes (TWAS-significant genes are provided in Supplemental Tables 3 and 4). We performed GO enrichment analysis of these genes and found that they were mostly enriched in glycolipid transport binding, transcriptional regulation and proline metabolism (not included in the manuscript). Interestingly, multiple homologs of ABC transporter family members (ABCC3) were identified in these negative genes, while phospholipid-transporting ATPase (ALA10) and fatty acid reductase (FAR6) were also associated in the positive genes. These results suggested that there may also be a potential correlation between seed coat development and oil content. We just marked genes involved in involved in the phenylpropanoid biosynthesis pathway and secondary cell wall development. None of the negatively associated genes (except TCH4) are involved in phenylpropanoid biosynthesis pathway and secondary cell wall development, so we did not mark the negative genes (except TCH4).*

*In addition, we added a column of Beta values in Supplementary Table 3 and 4, respectively, and if the Beta value is minus, it means that these genes were negatively associated with SCC.*

5. Is the gene CCR-LIKE an unreported gene? Can you add some gene evolutionary tree analysis and analysis of conserved structural domains of the gene? You should compare the CCR-LIKE and CCR in B. napus.

*Response 5: We found that CCRL is not a well-characterized gene and there is no functional study on this gene in Arabidopsis, B. napus or other species. We have performed the following analysis of the CCRL gene according to your suggestion.*

*(1) CCRL basic information analysis.*

*B. napus (Darmon) ID: BnaC07g42660D*

*B. napus (ZS11) ID: BnaC07G0497400ZS*

*Arabidopsis ID: AT4G30470*

*We first searched the homologs of BnaC07G0497400ZS in B. napus using BnTIR (http://yanglab.hzau.edu.cn/BnTIR). There are four homologous genes of BnaC07G0497400ZS in ZS11*.

*(2) CCRL and CCR family protein sequence alignment and phylogenetic tree analysis.*

*The results from protein sequence alignment analysis showed that CCRL shared less than 40% similarity with CCR, but CCRL shared conserved domain and amino acids with CCR (Fig. S8). We applied MEGA-X to perform phylogenetic tree analysis of the protein sequences. Bootstrap value of 1000 was used to test the confidence of the calculated evolutionary tree branches. It was found that CCRL was more related to CCR1 in B. napus (Fig. S9), and we have also in line 270-273.*

*(3) Conserved domain analysis of CCR-LIKE and CCR family genes.*

*We applied InterPro (http://www.ebi.ac.uk/interpro/) to analyze the conserved domains and found that both CCRL and CCR had NAD(P)-bd_dom_sf domain (IPR036291) and Epimerase_deHydtase domain (IPR001509), although they only showed less than 40% protein sequence similarity. Moreover, we also found that BnaCCRL had a reductase activity like CCR when p-coumaroyl-CoA, sinapoyl-CoA or feruloyl-CoA was used as a substrate (Fig. S10).*

6. The regulatory network of key genes for carbon source allocation is shown in Fig6c. How many of the SOC-TWAS significant associated genes are regulated by the A09 hotspot?

*Response 6: Thanks for your suggestion, we identified 403 TWAS-SOC significant genes, and 281 (69.73%) of them were regulated by the A09 hotspot.*

7. TWAS significant genes in eGWAS localized to the A09 region are mainly enriched in many categories related to phenylpropanoid metabolism and abiotic stresses". What are the features of other genes that are not localized to the A09 region? Are they enriched in some other pathways?

*Response 7: As you mentioned, we performed GO enrichment analysis for TWAS-SOC significant genes that eGWAS were not localized at the A09 hotspot region. We found that these genes are mostly enriched in prolyl metabolism, response to oxidative stress and enzyme activity pathways, while some were also enriched in flavonoid and secondary cell wall biosynthesis pathways.*

8. Is it possible to predict some key genes or transcription factors that regulate genes related to seed coat synthesis in the A09 hotspot region?

*Response 8: Thank you for your suggestion. We used a gene prioritization method to predict the candidate genes in the qSCC.A09 region using the POCKET package [3]. The results revealed that MYB52 involved in the regulation of secondary cell wall and mucilage synthesis in seed coat [4, 5] may be a key gene affecting SCC. MYB52 binding motifs were also found in the 2 kb upstream region of BnaTT8s. These results indicate that MYB52 in the qSCC.A09 region might control SCC by regulating the expression of BnaTT8s. But we have not provided the prediction of MYB52 in this manuscript because we are currently working on the function of MYB52 in the qSCC.A09 region (our future study). If any researchers are interested in this QTL, they would also be able to look at these genes in the QTL region.*

9. The oil in rapeseeds is mostly concentrated in the embryo, is there any data on the ratio of embryo to seed weight in the population, can you do some analysis that may be more related data on carbon source allocation between SCC and ratio of embryo to seed weight rather than SOC?

*Response 9: Thank you for your suggestion. However, because the seed embryos were easier to be damaged during the stripping process and not easy to be collected, we don't have this phenotype data for further study.*

Minor points:

1. The names of TT8 and CCRL should be highlighted in Fig2e.

*Response 1: Thank you for your suggestion. We have highlighted TT8 and CCRL in Fig2e.*

2. Fig3a should be clearly labeled with 3 significant QTLs and their names.

*Response 2: Thank you for your suggestion. We have clearly labeled the 3 significant QTLs in Fig3a.*

3. The SNP markers in Fig3c should be marked with more distinctive colors and the eQTLs should be clearly labeled as to which gene corresponds to the eQTL.

*Response 3: As suggested, we have clearly labeled the gene eQTL in Fig3c. However, the color of the marker SNP represents LD (R2), which is not easy to change, so we marked this SNP as an inverted triangle and enlarged it for making it more distinctive.*

4. Whether a histogram can be drawn for the hotspot regulatory regions identified in the whole genome, instead of just a dot plot.

*Response 4: Thank you for your suggestion. We have provided a histogram to better show the distribution of the eQTLs (Fig. S3).*

5. Since the modules identified in the study that were significantly negatively correlated with SCC, the correlation analysis between them and SOC should be added.

*Response 5: Thank you for your suggestion. M65 and M139 are associated with SOC which have already been reported in our previous study [3]. So we did not include the correlation analysis between them and SOC in this study.*

*Reference:*

*1. Zhai Y, Yu K, Cai S, Hu L, Amoo O, Xu L, Yang Y, Ma B, Jiao Y, Zhang C: Targeted mutagenesis of BnTT8 homologs controls yellow seed coat development for effective oil production in Brassica napus L. Plant biotechnology journal 2020, 18:1153-1168.*

*2. Baudry A, Heim MA, Dubreucq B, Caboche M, Weisshaar B, Lepiniec L: TT2, TT8, and TTG1 synergistically specify the expression of BANYULS and proanthocyanidin biosynthesis in Arabidopsis thaliana. The Plant Journal 2004, 39:366-380.*

*3. Tang S, Zhao H, Lu S, Yu L, Zhang G, Zhang Y, Yang Q-Y, Zhou Y, Wang X, Ma W, et al: Genome- and transcriptome-wide association studies provide insights into the genetic basis of natural variation of seed oil content in Brassica napus. Molecular Plant 2021, 14:470-487.*

*4. Shi D, Ren A, Tang X, Qi G, Xu Z, Chai G, Hu R, Zhou G, Kong Y: MYB52 Negatively Regulates Pectin Demethylesterification in Seed Coat Mucilage. Plant Physiol 2018, 176:2737-2749.*

*5. Zhong R, Lee C, Zhou J, McCarthy RL, Ye ZH: A battery of transcription factors involved in the regulation of secondary cell wall biosynthesis in Arabidopsis. Plant Cell 2008, 20:2763-2782.*

**Second round of review**

**Reviewer 1**

The revision looks all good to me.

**Reviewer 2**

I have read the revised manuscript which is much improved. It is very interesting that CCRL shares less than 40% homology with CCR, but CCRL had reductase activity as CCR, which provides direct evidence that CCRL is involved in lignin synthesis. This is a novel discovery. Authors claim this is the first time to report CCRL's enzyme activity and function, I suggest authors confirm it. It is OK to me that authors choose not to show the prediction of MYB52 as candidate gene in A09 hotspot region.

**Authors Response**

**Point-by-point responses to the reviewers’ comments:**

Reviewer 2#:

I have read the revised manuscript which is much improved. It is very interesting that CCRL shares less than 40% homology with CCR, but CCRL had reductase activity as CCR, which provides direct evidence that CCRL is involved in lignin synthesis. This is a novel discovery. Authors claim this is the first time to report CCRL's enzyme activity and function, I suggest authors confirm it. It is OK to me that authors choose not to show the prediction of MYB52 as candidate gene in A09 hotspot region.

*Response: we did literature and database search again. It is confirmed that our study is the first report on CCRL’s biochemical activity and biological function involved in lignin biosynthesis in plants.*
